# Charge-Transfer Interactions Stabilize G-Quadruplex-Forming Thrombin Binding Aptamers and Can Improve Their Anticoagulant Activity

**DOI:** 10.3390/ijms22179510

**Published:** 2021-09-02

**Authors:** Kévan Pérez de Carvasal, Claudia Riccardi, Irene Russo Krauss, Domenico Cavasso, Jean-Jacques Vasseur, Michael Smietana, François Morvan, Daniela Montesarchio

**Affiliations:** 1 Institut des Biomolécules Max Mousseron, University Montpellier, CNRS, ENSCM, 34095 Montpellier, France; kevan.perez-de-carvasal@etu.umontpellier.fr (K.P.d.C.); jean-jacques.vasseur@umontpellier.fr (J.-J.V.); michael.smietana@umontpellier.fr (M.S.); 2 Department of Chemical Sciences, University of Naples Federico II, Via Cintia 21, I-80126 Naples, Italy; claudia.riccardi@unina.it (C.R.); irene.russokrauss@unina.it (I.R.K.); domenico.cavasso@unina.it (D.C.); 3 CSGI—Consorzio Interuniversitario per lo Sviluppo dei Sistemi a Grande Interfase, Via della Lastruccia 3, I-50019 Sesto Fiorentino, Italy

**Keywords:** thrombin binding aptamer, G-quadruplex, 1,5-dialkoxy naphthalene (DAN), 1,8,4,5-naphthalenetetra-carboxylic diimide (NDI), charge transfer, oligonucleotide, anticoagulant activity

## Abstract

In the search for optimized thrombin binding aptamers (TBAs), we herein describe the synthesis of a library of TBA analogues obtained by end-functionalization with the electron-rich 1,5-dialkoxy naphthalene (DAN) and the electron-deficient 1,8,4,5-naphthalenetetra-carboxylic diimide (NDI) moieties. Indeed, when these G-rich oligonucleotides were folded into the peculiar TBA G-quadruplex (G4) structure, effective donor–acceptor charge transfer interactions between the DAN and NDI residues attached to the extremities of the sequence were induced, providing pseudo-cyclic structures. Alternatively, insertion of NDI groups at both extremities produced TBA analogues stabilized by π–π stacking interactions. All the doubly-modified TBAs were characterized by different biophysical techniques and compared with the analogues carrying only the DAN or NDI residue and unmodified TBA. These modified TBAs exhibited higher nuclease resistance, and their G4 structures were markedly stabilized, as evidenced by increased T_m_ values compared to TBA. These favorable properties were also associated with improved anticoagulant activity for one DAN/NDI-modified TBA, and for one NDI/NDI-modified TBA. Our results indicated that TBA pseudo-cyclic structuring by ad hoc designed end-functionalization represents an efficient approach to improve the aptamer features, while pre-organizing and stabilizing the G4 structure but allowing sufficient flexibility to the aptamer folding, which is necessary for optimal thrombin recognition.

## 1. Introduction

Nucleic acid-based aptamers represent a promising class of short DNA or RNA molecules which, upon folding into peculiar three-dimensional arrangements, act as specific and high-affinity ligands for a wide range of biologically relevant targets [1,2,3]. Starting from large pools of random-sequence oligonucleotides, aptamers are generally identified through a reiterative fishing procedure based on affinity chromatography, known as SELEX (systematic evolution of ligands by exponential enrichment) [4,5,6,7,8]. Notably, aptamers exhibit several advantages compared to antibodies [1,2], and, for this reason, have gained interest as agents of choice in both diagnostic [9,10,11] and therapeutic [12,13,14,15,16,17] applications. However, the biomedical use of aptamers still proceeds slowly; just a few candidates have entered clinical trials [14,18,19,20].

The practical applications of aptamers are primarily limited by their susceptibility to nuclease degradation, rapid blood/renal elimination or suboptimal thermal stability. In this context, the post-SELEX modification of aptamers is a valuable strategy to improve their features and to obtain more effective therapeutic agents [18,21,22,23,24,25,26,27]. Chemical modifications of nucleic acids considerably increase the functional and structural diversity of aptamer libraries and substantially improve their target affinities and/or biological properties. Additionally, chemically modified nucleic acid aptamers exhibit much higher resistance to in vivo nuclease action [22,23,25,26,27,28,29,30].

The oligonucleotide aptamer which has been most investigated, and also claims the highest number of structural variants thus far synthesized, is the 15-mer thrombin binding aptamer, also known as TBA (5′-GGTTGGTGTGGTTGG-3′) [31,32]. This G-rich oligonucleotide interferes with the coagulation cascade, selectively recognizing the fibrinogen-binding exosite I of thrombin [33,34,35], a multifunctional “trypsin-like” serine protease with a pivotal function in the last step of blood clotting [36,37,38,39]. Previous structural studies demonstrated that TBA adopts a chair-like, antiparallel, unimolecular G-quadruplex (G4) structure both in the free and protein-bound form [34,35,40]. Although TBA evidenced a promising pharmacokinetic profile in humans, its preclinical and clinical evaluations were halted after phase I studies due to suboptimal dosing profiles [41,42,43]. Thus, to improve its biophysical and biological properties, a large number of TBA analogues have been proposed [31,32].

In this context, our research group recently explored a cyclization approach [44,45] to develop optimized TBA analogues. By chemically linking the 5′ and 3′ extremities of the oligonucleotide using different flexible linkers, several cyclic TBA analogues were obtained [46,47]. Fine-tuning of the length and chemical nature of the linker allowed identifying one cyclic derivative with improved enzymatic resistance in serum and a dramatically stabilized G4 structure associated with enhanced anticoagulant activity compared to the unmodified aptamer [47]. This approach was then extended to NU172, another anticoagulant aptamer [48,49], with which we varied the length and chemical properties of the linker connecting the extremities of the G4 domain, thereby obtaining a focused set of cyclic NU172 derivatives. However, in all cases, a lower inhibitory activity than that of the unmodified aptamer was obtained [50]. These outcomes suggested that increased rigidity of the G4 structure—both in TBA and NU172—as a consequence of the cyclic structure, could somehow reduce the aptamer’s affinity for the protein, and thus its bioactivity. Our previous results further highlighted the need to realize a better compromise between pre-organizing/stabilizing the TBA G4 structure and maintaining some structural flexibility, which is crucial for thrombin recognition.

Thus, to expand the structural diversity of TBA derivatives, we took inspiration from a recent work by some of us [51], and in the current study, we developed novel modified TBA analogues end-functionalized with phosphodiester-linked donor–acceptor pairs based on the electron-rich 1,5-dialkoxy naphthalene (DAN) and electron-deficient 1,8,4,5-naphthalenetetra-carboxylic diimide (NDI) moieties. Our idea was that donor–acceptor interactions [52], also known as charge transfer interactions [53], realized by the DAN and NDI residues attached to the termini of the TBA sequence, can be established only when the oligonucleotide is folded into its peculiar antiparallel G4 architecture, thereby providing a non-covalent cyclic-like structure. This structuring, occurring in the presence of the target thrombin or of suitable cations, should guarantee the G4 flexibility necessary to recognize the biological target, accompanied by the exceptional advantages found for the previously investigated cyclic TBAs [46,47], i.e., higher G4 thermal stability and improved nuclease resistance.

**Figure 1:**
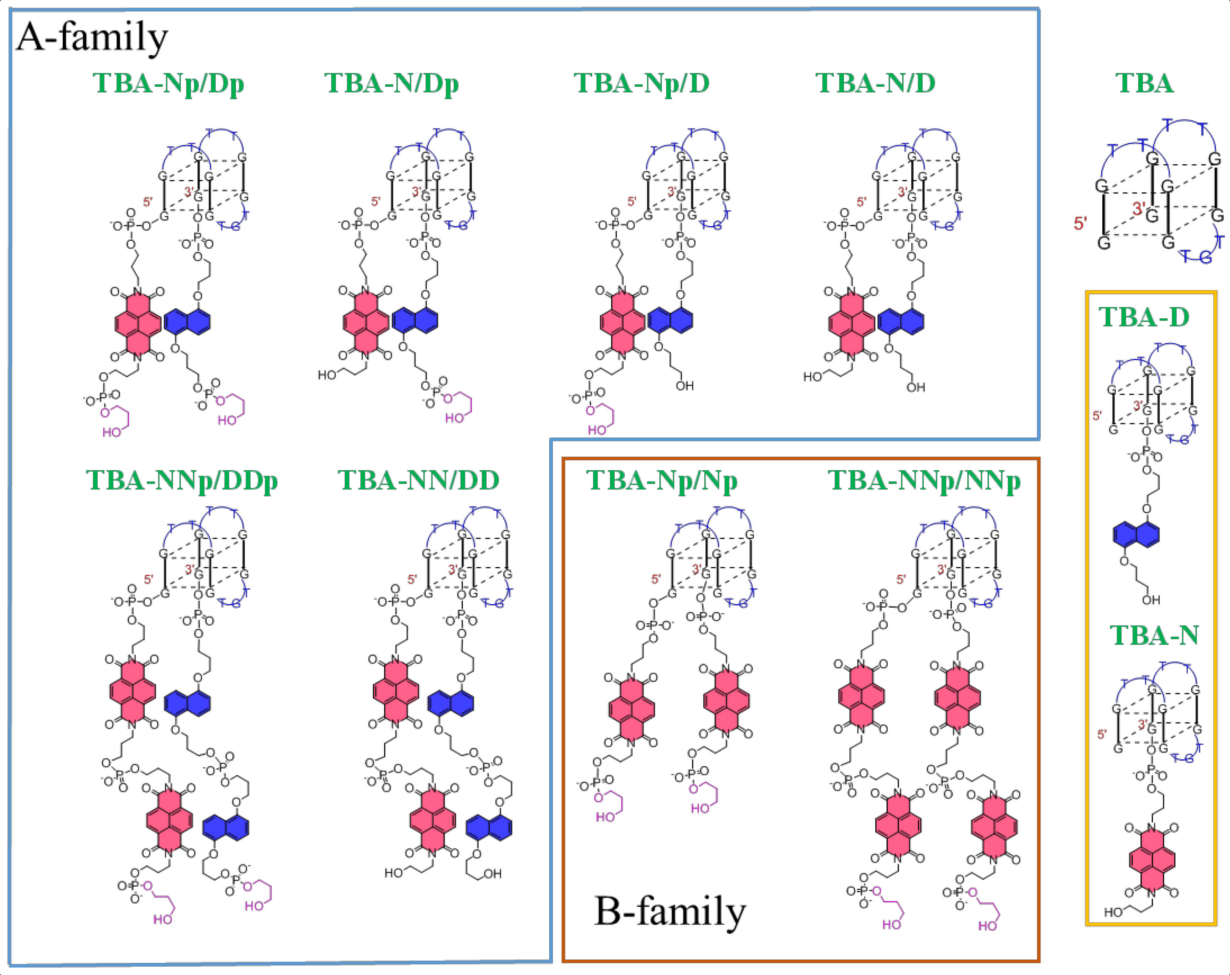
Chemical structures of the oligonucleotides investigated herein. N = naphthalene diimide; D = dialkoxy naphthalene; p = 1,3-propanediol phosphate.

Herein, we report the design, synthesis and biophysical/biological characterization of novel TBA derivatives, for which the TBA sequence was conjugated at its 5′ and 3′ ends with NDI (N) and DAN (D) units, respectively, and a terminal 1,3-propanediol monophosphate group (p) was provided to some, thereby leading to TBA analogues with one or two N/D pairs (**TBA-Np/Dp**, **TBA-N/Dp**, **TBA-Np/D**, **TBA-N/D**, **TBA-NNp/DDp** and **TBA-NN/DD**, A-family, Figure 1). The terminal 1,3-propanediol monophosphate group (p) was introduced to some analogues to partially counterbalance the lipophilic contribution to the aptamer given by the NDI and DAN residues and thus vary the overall hydrophilic/lipophilic effects in the investigated TBA series. Then, for comparison, only NDI moieties were introduced at both extremities, providing TBA analogues with one or two N/N pairs (**TBA-Np/Np** and **TBA-NNp/NNp**, B-family; Figure 1). Furthermore, the derivatives carrying at their 3′ ends a single DAN (**TBA-D**) or NDI (**TBA-N**) were also prepared and studied as reference compounds (Figure 1). In the A-family of the investigated TBA analogues, the G-quadruplexes are stabilized by charge transfer interactions that are an enhanced variant of π–π stacking [53]. In the B-family, the G4 structures are stabilized by classical π–π stacking. TBA analogues with only DAN at both ends were not considered, since it is already known that a D/D pair causes destabilization [54].

The influences of the different conjugating groups on the conformational behavior, thermal stability and nuclease resistance of the modified TBAs were investigated in comparison with the unmodified aptamer. Finally, light scattering (LS) analyses were carried out to determine the anticoagulant activity levels of the novel analogues.

With the design of this library of pseudo-cyclic TBAs, we intended to obtain effective analogues with high potency for inhibiting fibrin clot formation, concomitantly featuring increased G4 thermal stability and resistance to nucleases.

## 2. Results and Discussion

### 2.1. Solid Supported Synthesis of NDI and DAN TBA Analogues and Their Characterization

**Scheme 1:**
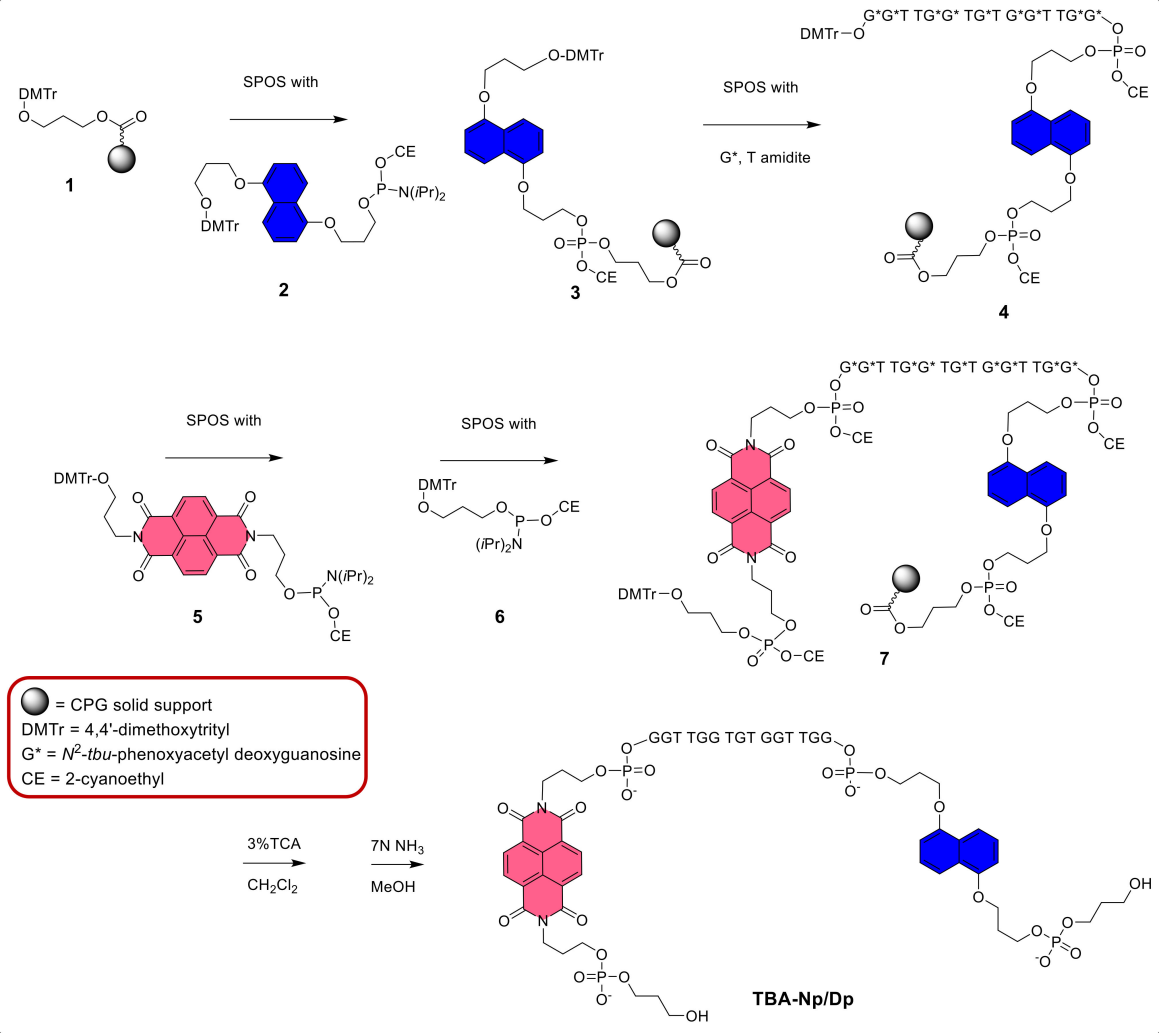
Solid-phase oligonucleotide synthesis (SPOS) of **TBA-Np/Dp**.

All oligonucleotides were prepared via solid phase synthesis according to standard phosphoramidite chemistry [55] using the *tert*-butyl phenoxyacetyl protecting group for the deoxyguanosine phosphoramidite building blocks and Q-Linker CPG supports [56] in the place of the standard succinyl linker in order to avoid a final strong basic treatment for the removal of the oligonucleotides from the solid support that could have destroyed the diimide function of the NDI residues. As a representative example, Scheme 1 reports the synthesis of the **TBA-Np/Dp** analogue. Using a DNA synthesizer and starting from 1,3-propanediol-functionalized solid support **1**, the DAN phosphoramidite **2** was coupled. Then, the TBA sequence was assembled followed by coupling with NDI **5** and propanediol **6** phosphoramidites [51]. After detritylation, treatment with methanolic ammonia afforded the expected **TBA-Np/Dp** analogue, which was analyzed and purified by RP-HPLC and then characterized by MALDI-TOF mass spectrometry. The other TBA analogues were synthesized following similar procedures starting from ad hoc functionalized solid supports, as indicated in Schemes S1–S3.

To check the purity of the synthesized TBA analogues, a 20% denaturing PAGE analysis was carried out, in comparison with unmodified TBA (Figure S1). Under these denaturing conditions, all the investigated oligonucleotides migrated as single bands on the gel, showing only slight differences in their electrophoretic mobility depending on their different mass/charge ratios.

### 2.2. NDI and DAN Ends Confer Higher Thermal Stability to TBA

#### 2.2.1. Conformational and Spectroscopic Properties of the Modified TBA Analogues

The spectroscopic properties and conformational behavior of the modified TBAs were examined by UV and CD analyses, in comparison with the unmodified aptamer. In order to evaluate the effects of different saline conditions on the structuring ability of the investigated TBA analogues, these studies were performed using two different phosphate buffer solutions, containing a high content of K^+^ (10 mM KH_2_PO_4_/K_2_HPO_4_, 70 mM KCl, 0.2 mM EDTA, pH 7.3, here indicated as K^+^-rich buffer) or of Na^+^ (i.e., PBS: 137 mM NaCl, 2.7 mM KCl, 10 mM NaH_2_PO_4_/Na_2_HPO_4_, 1.8 mM KH_2_PO_4_/K_2_HPO_4_, pH 7.3, here indicated as Na^+^-rich buffer) ions, mimicking the intra- and extracellular environments. In fact, buffer composition, and particularly ion concentration, plays a key role in G4 folding, determining dramatic changes in the biophysical [35,57] and biological properties of TBA [57,58,59,60].

##### 2.2.1.1. UV Spectroscopy Analysis

**Figure 2:**
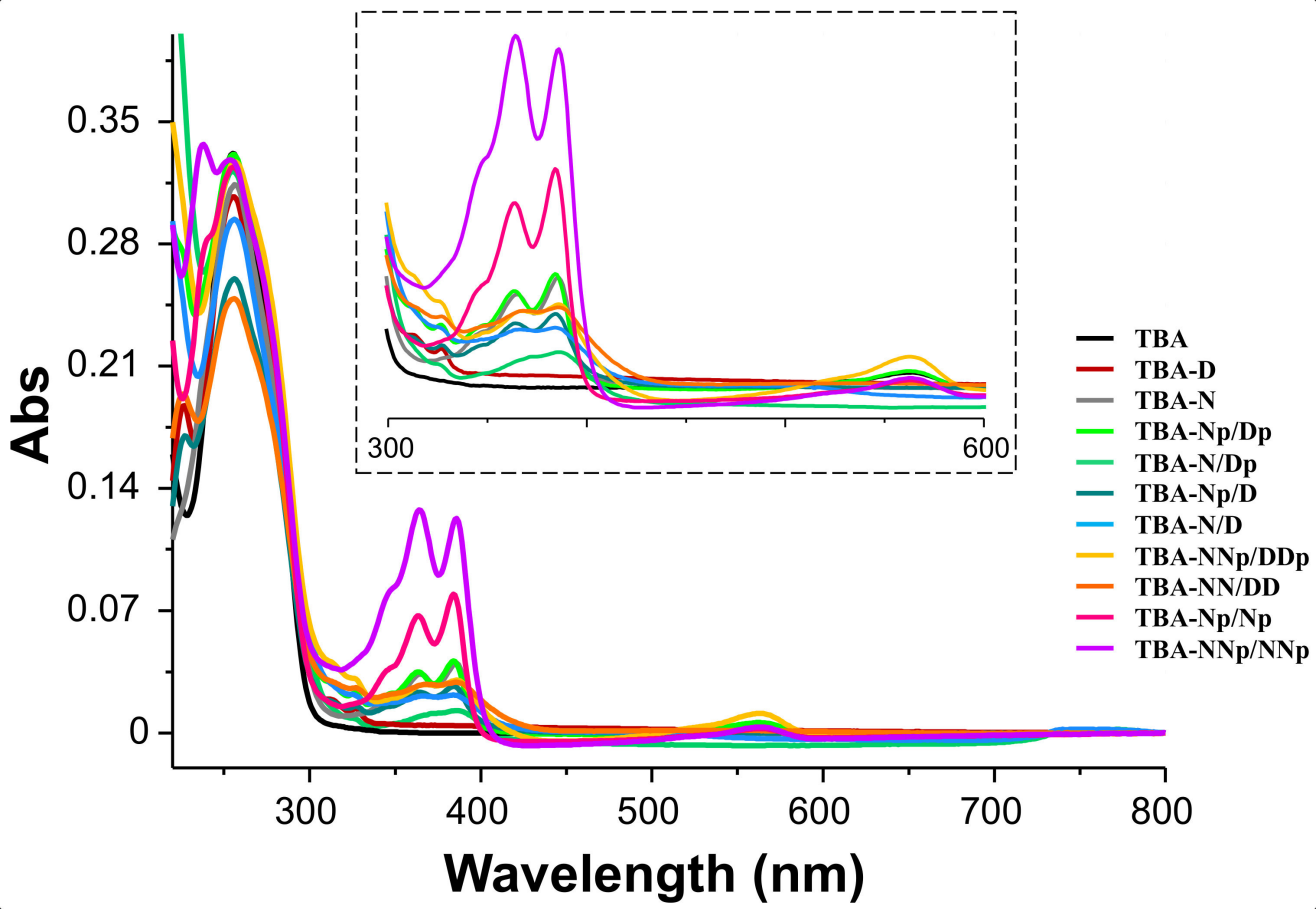
Overlapping UV-vis spectra of the pseudocyclic TBA derivatives in comparison with unmodified TBA, TBA-D and TBA-N, used as reference sequences. All the investigated oligonucleotides were analyzed at 2 µM in H_2_O at room temperature. The inset shows an enlargement of the 300–600 nm region of the spectra, to better highlight the characteristic UV-vis contribution of naphthalene diimide groups and the charge transfer band around 560 nm or the NDI exciplex band.

First, UV-vis absorption spectra of the TBA derivatives were recorded in H_2_O at room temperature in comparison with TBA, **TBA-D** and **TBA-N**, which were used as reference sequences (Figure 2). These spectra revealed the characteristic absorptions of TBA—the double-hump band between 230 and 300 nm [46]; and the NDI probe—UV-vis contribution in the 300–600 nm region, as previously found [51]. In detail, the oligonucleotides conjugated with the NDI appendages exhibited main absorption bands centered at 364 and 386 nm, due to π–π* transitions, as found for the reference **TBA-N**. An additional charge transfer band centered at 562 nm was found, especially for the oligonucleotide carrying two DAN and two NDI moieties. These spectral features are consistent with those found for the previously studied (DAN-NDI)_3_ hexamer and confirm the tight interaction between the terminal appendages of the TBA sequence [51]. Likewise, the TBA analogue with four NDI moieties exhibited a well-defined exciplex band centered at around 560 nm. In contrast, the presence of a single DAN group provided a poorly detectable absorption band in the 290–320 nm range. Remarkably, as highlighted from the comparison with unmodified TBA analyzed under the same experimental conditions, the contributions to the 260 nm absorbance of the NDI and DAN moieties could be considered negligible for all the modified TBAs.

**Figure 3:**
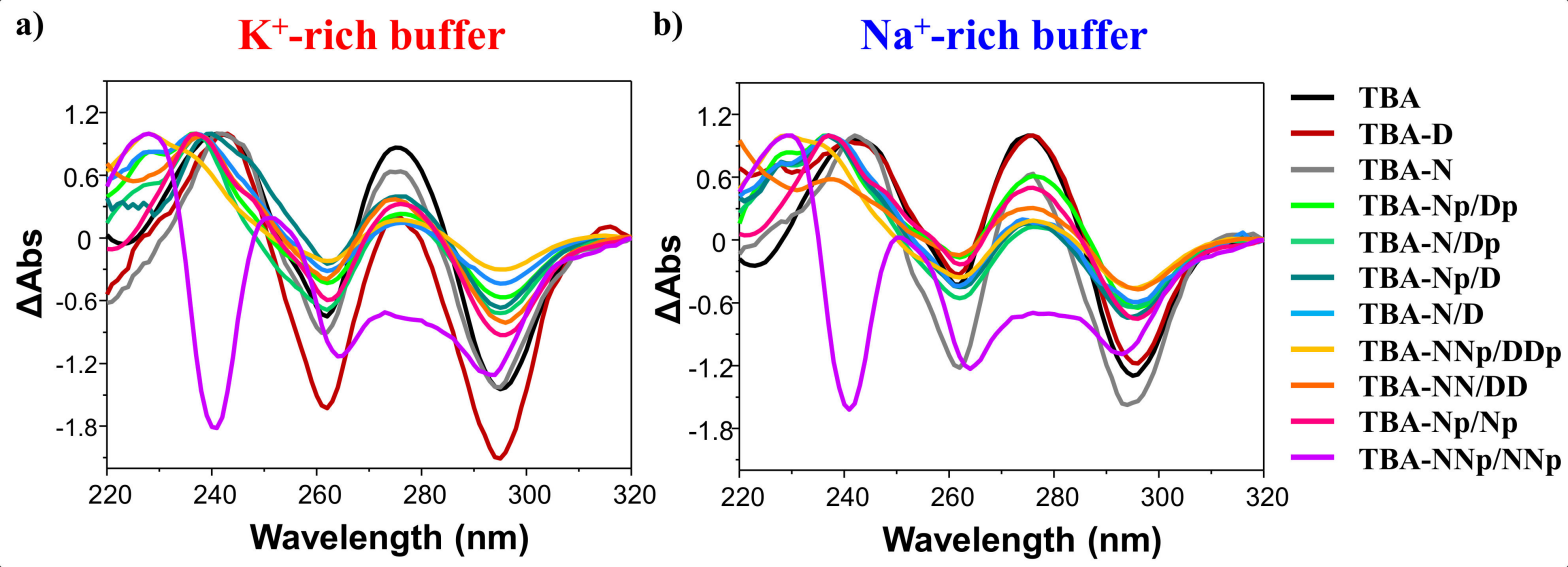
Representative normalized TDS profiles of the TBA analogues investigated at 2 µM in both the K^+^-rich (**a**) and Na^+^-rich (**b**) buffer solutions, in comparison with unmodified TBA (black line). TDS profiles resulted from the subtraction of each UV spectrum registered at 15 °C from the corresponding 90 °C one.

Then, UV thermal difference spectra (TDS, Figure 3) were obtained for all the investigated oligonucleotides in the selected buffer solutions. The UV spectra were recorded at 2 μM at low and high temperatures (i.e., 15 and 90 °C). The UV spectral difference between the unfolded and folded G4-forming sequence represents indeed a “fingerprint” of a selected nucleic acid structure and particularly allows identifying the presence in solution of a G4 architecture [61,62,63,64].

In both K^+^- and Na^+^-rich solutions, all the TBA analogues investigated exhibited normalized TDS profiles featuring two positive (at ca. 240 and 275 nm) and two negative bands (around 260 and 295 nm), which are similar to those of unmodified TBA (Figure 3, black line) and are characteristic of G4 structure formation [61,62,63,64].

The only exception to this general behavior was represented by the **TBA-NNp/NNp** derivative (Figure 3, violet line), whose TDS profile, albeit maintaining the maximum and minimum, respectively, around 270 and 295 nm, showed positive values at ca. 230 and 250 nm and strong negative absorbance around 240 nm. These spectral characteristics could not be unambiguously associated with any well-defined nucleic acid structure and could be indicative of significant polymorphism [61].

In addition, an estimation of the G4 folding predominant in solution was obtained by calculating the TDS factors. In detail, ΔA_240_/ΔA_295_, ΔA_255_/ΔA_295_ and ΔA_275_/ΔA_295_ TDS factors were determined, and the results indicated values lower than 2, 1.5 and 2, respectively (Table S1), which are all consistent with an antiparallel G4 topology [63]. Notably, also for **TBA-NNp/NNp**, all three TDS factor values were perfectly in line with those of a typical antiparallel G4 conformation (Table S1).

Taken together, these results demonstrated that the conjugation with DAN and/or NDI moieties had no dramatic influence on the overall antiparallel G-quadruplex folding of the TBA sequence.

##### 2.2.1.2. CD Spectroscopy Analysis: CD Spectra and CD Thermal Denaturation/Renaturation Measurements

**Figure 4:**
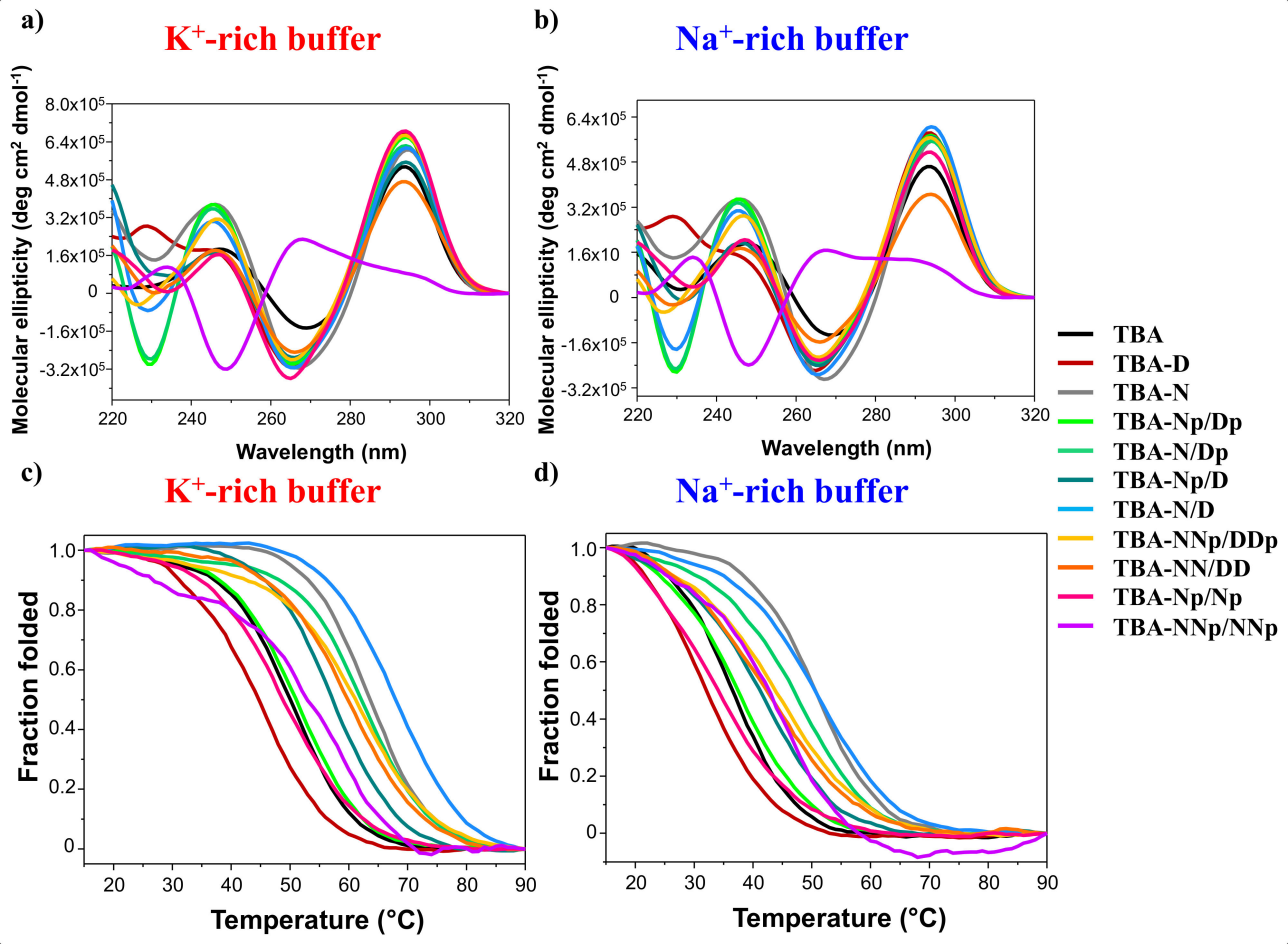
Representative overlapping CD spectra (**a**,**b**) and CD-melting profiles (**c**,**d**) of the TBA analogues investigated in comparison with unmodified TBA (black line). CD spectra were recorded at 2 µM and 15 °C in both the K^+^-rich (**a**) and Na^+^-rich (**b**) buffer solutions. CD-melting profiles—at 2 µM in the 15–90 °C range using a scan rate of 1 °C/min—were recorded in both the K^+^-rich (**c**) and Na^+^-rich (**d**) buffer solutions at the maximum ellipticity observed for each oligonucleotide system and are reported as folded fractions of each oligonucleotide system as a function of temperature.

In order to deeply investigate the effects of the DAN and/or NDI appendages on the conformational properties of the G4 structure, CD spectra of the TBA analogues—at 15 °C and 2 µM oligonucleotide concentration—were recorded in comparison with their natural counterparts (Figure 4, panels a and b, respectively, for the K^+^- and Na^+^-rich buffers).

In both saline conditions, all the modified TBAs showed CD spectra essentially superimposable on that of the native aptamer (Figure 4, black line), with similar amplitudes for the main CD signals—i.e., two positive bands, with maxima at about 295 and 247 nm, and one negative band with minima at 268 nm. These spectroscopic properties are fully consistent with an antiparallel G4 topology in which *anti* and *syn* guanosines alternate along the strands [63,65,66,67,68,69,70,71,72].

Only the **TBA-NNp/NNp** analogue showed a completely different CD profile in both the analyzed buffer solutions, with a main ellipticity centered at 268 nm and negative CD values at around 249 nm. In order to get deeper insights into the G4 topology adopted by **TBA-NNp/NNp** in solution, the CD spectra were also processed by singular value decomposition (SVD) analysis [72]. The use of the software developed by del Villar-Guerra et al. gave fitted CD profiles in poor accordance with the experimental CD curves of **TBA-NNp/NNp** (Figure S2). This finding, coupled with the peculiar behavior in both UV and CD measurements, suggested that a marked polymorphism could be operative for this TBA analogue. This could probably include multiple G4 conformations and also alternative nucleic acid structures in solution, while maintaining a prevalent antiparallel G4 conformation, as revealed by the outcomes of the TDS factor. These differences could be associated with additional stacking interactions due to the presence of four NDI moieties.

For all the investigated oligonucleotides, positive CD bands showed higher molar ellipticities in K^+^ than in Na^+^-containing media, according to the well-known dependence of G4 stability from the main cations present in the saline solution (Figure 4) [71,73].

Differences in the behavior of the TBA analogues are clearly evident by comparing their CD-thermal denaturation/renaturation profiles, obtained by monitoring the CD signal—at the maximum ellipticity for each oligonucleotide system—while varying the temperature. For these experiments, all the oligonucleotides were analyzed at 2 µM in both the K^+^- and Na^+^-rich media in the 15–90 °C range, in comparison with unmodified TBA. The overlapping CD-melting profiles of all the studied oligonucleotides are reported in terms of folded fraction as functions of the temperature in Figure 4 (panels c and d, respectively, for the K^+^- and Na^+^-rich buffers). The overlapping CD-melting/annealing profiles for each compound are shown in Supplementary Figures S3–S13, along with the superimpositions of the spectra recorded at 5 °C steps during each heating and cooling experiment. An overview of the apparent T_m_ values derived by CD-monitored thermal denaturation/renaturation measurements is reported in Table 1.

In both the investigated buffer solutions, all the TBA analogues—except **TBA-D** and **TBA-Np/Np**—revealed notably increased thermal stability compared to unmodified TBA, indicating the formation of very stable G4 structures, although exhibiting slightly different trends based on the tested saline conditions (Figure 4 and Table 1).

In detail, in the selected K^+^-rich buffer, the most stabilized G4 structures were those of **TBA-N/D**, **TBA-N**, **TBA-N/Dp**, **TBA-NNp/DDp** and **TBA-NN/DD**, which showed apparent T_m_ values of 68, 64, 62, 62 and 61 °C, corresponding to ΔT_m_ of +17, +13, +11, +11 and +10, respectively, compared to unmodified TBA. Slightly improved T_m_ values of 57 and 56 °C—with ΔT_m_ of +6 and +5 °C with respect to the native aptamer—were found for **TBA-Np/D** and **TBA-NNp/NNp**, respectively. On the other hand, **TBA-Np/Dp** showed similar G4 thermal stability to that of unmodified TBA, with the same apparent T_m_ values within the experimental error. Conversely, CD analysis on **TBA-Np/Np** and **TBA-D** analogues indicated a slight destabilization of their G4 structures with respect to unmodified TBA, with T_m_ values, respectively, of 49 and 45 °C, i.e., 2 and 6 °C lower than the native aptamer (Figure 4 and Table 1).

A similar pattern was observed in the Na^+^-rich medium. **TBA-N/D**, **TBA-N**, **TBA-N/Dp**, **TBA-NNp/DDp** and **TBA-NNp/NNp** formed the most stable G4 structures with apparent T_m_ values of 51, 51, 48, 44 and 44 °C, corresponding to ΔT_m_ of +15, +15, +12, +8 and +8, respectively, compared to the native TBA. A lower increase in T_m_ values was found for **TBA-Np/D** and **TBA-NN/DD**, both exhibiting T_m_ values of 42 °C, i.e., 6 °C higher than unmodified TBA. Additionally, **TBA-Np/Dp** showed a slight stabilization compared to the native sequence, with a T_m_ value of 38 °C (ΔT_m_ = +2). Finally, **TBA-Np/Np** and **TBA-D** proved to be the worst analogues of the series, with ΔT_m_ of −2 and −5 °C with respect to unmodified TBA (Figure 4 and Table 1).

Notably, in the Na^+^-rich buffer, the apparent T_m_ value recorded for **TBA-Np/Dp** was roughly equal to 37 °C, and both **TBA-Np/Np** and **TBA-D** were poorly folded at physiological temperature (Figure 4 and Table 1).

Taken together, the CD-derived T_m_ values revealed these trends of G4 stability: **TBA-N/D** > **TBA-N** > **TBA-N/Dp** ≈ **TBA-NNp/DDp** > **TBA-NN/DD** > **TBA-Np/D** > **TBA-NNp/NNp** > **TBA-Np/Dp** ≈ TBA > **TBA-Np/Np** > **TBA-D** in the K^+^-rich buffer; and **TBA-N/D** ≈ **TBA-N** > **TBA-N/Dp** > **TBA-NNp/DDp** ≈ **TBA-NNp/NNp** > **TBA-NN/DD** ≈ **TBA-Np/D** > **TBA-Np/Dp** ≈ TBA > **TBA-Np/Np** > **TBA-D** in the Na^+^-rich buffer solution (Table 1). The data are in accordance with the general finding that G4 structures are more stable in K^+^- (ΔT_m_ ~ +15 °C) than in Na^+^-rich solutions [71,73,74].

Notably, in Na^+^-rich buffer, with respect to TBA-N/D, which does not have propanediol phosphate groups at its ends, the introduction of one propanediol phosphate group at the 3′ end (**TBA-N/Dp**) led to a ΔT_m_ of −3 °C, whereas its introduction at the 5′ end led to an even higher destabilization (ΔT_m_ = −9 °C for **TBA-Np/D**). In turn, the insertion of two propanediol phosphate groups, one at each end of the oligonucleotide, led to an almost additive destabilization (ΔT_m_ = −13 °C for **TBA-Np/Dp**). A possible explanation for this behavior is the presence of an extra negative charge due to the additional phosphodiester linkage that induces a detrimental repulsive effect with the other phosphodiesters of the aptamer. When another NDI/DAN pair is added (**TBA-NN/DD**), the negative effect of the additional phosphodiester bonds is counterbalanced by the stabilization of the additive N/D pair, leading to a lower destabilization (ΔT_m_ of −9 °C compared to **TBA-N/D**). The insertion of additional propanediol phosphate groups for **TBA-NNp/DDp** has a lesser effect, possibly due to reduced phosphates repulsion as a consequence of an increased flexibility. A similar trend was observed also in the selected K^+^-rich buffer. The overall data confirm the slightly better stabilization induced by charge transfer interactions than by classical π–π stacking (cf. the higher T_m_ of **TBA-Np/Dp** than **TBA-Np/Np**). Interestingly, **TBA-N** exhibited strong stabilization (ΔT_m_ + ~14 °C) thanks to the noteworthy ability of NDIs to interact with terminal G-quartets of G-quadruplexes [75].

In all cases, the CD analysis on the studied TBA derivatives revealed nice sigmoidal profiles (Figure 4 and Supplementary Figures S3–S13), with no significant hysteresis between the heating and cooling profiles, thereby indicating that, under the experimental conditions used (temperature rate of 1 °C/min), the related denaturation/renaturation processes were reversible.

During each melting and annealing experiment, CD spectra—recorded in 5 °C steps (Supplementary Figures S3–S13)—denoted a notable and progressive reduction of the main CD bands upon increasing the temperature. These results suggested that all the modified TBAs behaved as the native aptamer from a qualitative point of view. In addition, independently from the buffer composition, all the modified TBAs were fully denatured at 90 °C and always completely recovered the initial spectral features after the heating/cooling cycle (Supplementary Figures S3–S13), further confirming the reversibility of the whole process. In particular, in both saline conditions, the superimposition of CD spectra obtained during thermal denaturation/renaturation experiments provided an isodichroic point at about 280 nm (Supplementary Figures S3–S13), consistent with a two-state process [76,77,78]. The only exception to this general behavior was found for **TBA-NNp/NNp** (Figure S13), suggesting that this TBA analogue did not unfold following a two-state process, in accordance with the hypothesized presence of a mixture of different G4 and non-G4 structures, as previously discussed.

Therefore, in order to determine their thermodynamic parameters, the CD-melting data of all the oligonucleotides—except **TBA-NNp/NNp**—were also elaborated according to the van’t Hoff analysis, based on a “two-state process” assumption [62,79,80]. The standard enthalpy and entropy change values of unmodified TBA in both the selected saline conditions were in good agreement with those reported in the literature under similar experimental conditions (Table S2) [46,47,81,82,83].

For all the TBA derivatives, analogously to their natural counterpart, aptamer folding is an enthalpy-driven process and the ΔH^0^ values are consistent with two G-tetrad-based G-quadruplex structures [84,85,86,87]. With the exception of **TBA-N**, all the investigated analogues are characterized by standard enthalpy and entropy changes lower in absolute values compared to unmodified TBA in both the analyzed saline conditions. The extra stability observed for **TBA-N** (Table S2) in both buffer solutions with respect to native TBA is mainly due to the enthalpic factor, suggesting the formation of additional interactions in the folded state, probably associated with a peculiar stacking of the NDI probe on the G4, as expected considering the well-known ability of ligands of this kind to profitably interact with G4 structures via end-stacking [75]. Where observed, the increased stability of the TBA derivatives with respect to unmodified TBA can be mainly attributed to a favorable ΔS^0^ change, which balances the unfavorable change of ΔH^0^, similarly to what was observed for the previously studied cyclic TBAs (Table S2) [46,47].

These energetic contributions can be rationalized considering the stacking interactions occurring between the electron acceptor/donor pair used for TBA functionalization, which, as expected, can pre-organize the corresponding G4 in a pseudo-cyclic structure. The whole set of thermodynamic parameters is consistent with the CD-derived T_m_ values and thus with the general G4 stability trend that was observed. These data were also confirmed by UV-monitored thermal denaturation/renaturation measurements performed at 295 nm (data not shown).

### 2.3. Non-Denaturing Polyacrylamide Gel Electrophoresis

**Figure 5:**
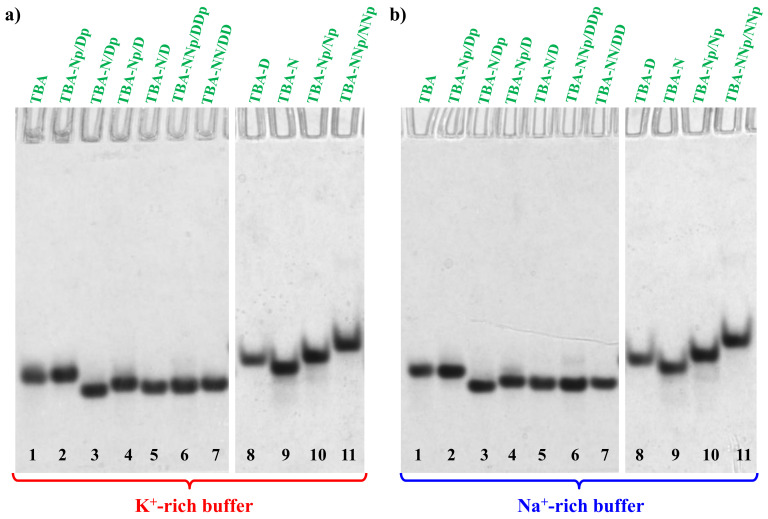
Representative 15% polyacrylamide gel electrophoresis under native conditions of the oligonucleotide samples at 15 μM in the selected K^+^-rich (**a**) and Na^+^-rich (**b**) buffer solutions, run at 80 V at r.t. for 2.5 h in TBE 1X buffer. Lane 1: TBA; lane 2: **TBA-Np/Dp**; lane 3: **TBA-N/Dp**; lane 4: **TBA-Np/D**; lane 5: **TBA-N/D**; lane 6: **TBA-NNp/DDp**; lane 7: **TBA-NN/DD**; lane 8: **TBA-D**; lane 9: **TBA-N**; lane 10: **TBA-Np/Np**; lane 11: **TBA-NN/pNNp**.

In order to further confirm that the investigated TBA analogues were able to preserve the monomolecular G4 structure typical of TBA, not forming aggregates or additional superstructures in solution [88,89], a native 15% polyacrylamide gel analysis was carried out (Figure 5). Electrophoretic experiments revealed that all the TBA derivatives in both phosphate buffer solutions migrated as single bands on the gel, indicating in all cases the presence of unimolecular species.

In detail, **TBA-N/Dp**, **TBA-Np/D**, **TBA-N/D**, **TBA-NNp/DDp** and **TBA-NN/DD** analogues (lanes 3–7, Figure 5) exhibited slightly increased electrophoretic mobility with respect to unmodified TBA, as previously observed for its cyclic derivatives [46,47]. As far as **TBA-Np/Dp** and **TBA-N** are concerned (lanes 2 and 9), these TBA variants showed similar migration abilities to their natural counterpart, whereas **TBA-D**, **TBA-Np/Np** and **TBA-NNp/NNp** (lanes 8, 10 and 11) migrated slightly slower than unmodified TBA.

Taking into account that the gel mobility of a G4 structures under non-denaturing conditions is mostly affected by its conformation and compactness, PAGE results suggested more compact G4 structures for **TBA-N/Dp**, **TBA-Np/D**, **TBA-N/D**, **TBA-NNp/DDp** and **TBA-NN/DD** analogues, and less compact G4 structures for **TBA-D**, **TBA-Np/Np** and **TBA-NNp/NNp** derivatives, with respect to unmodified TBA. Not surprisingly, **TBA-D** and **TBA-Np/Np**—which proved to be the least stable G4 structures, according to their CD-derived T_m_ values (Section 2.2.1.2)—showed scarcely compact G4 structures. In contrast, **TBA-NNp/NNp** (lanes 11) was the most retarded aptamer in this series, probably also as a result of its higher molecular weight than the other investigated TBAs, but, considering its polymorphism, its migration ability is difficult to rationalize. In all cases, no additional retarded band, attributable to large aggregates or higher-order G4 structures, was found, thereby indicating the exclusive formation of monomolecular G4 structures, analogously to unmodified TBA.

### 2.4. NDI and DAN Conjugating Groups Confer Higher Nucleases Stability to TBA

**Figure 6:**
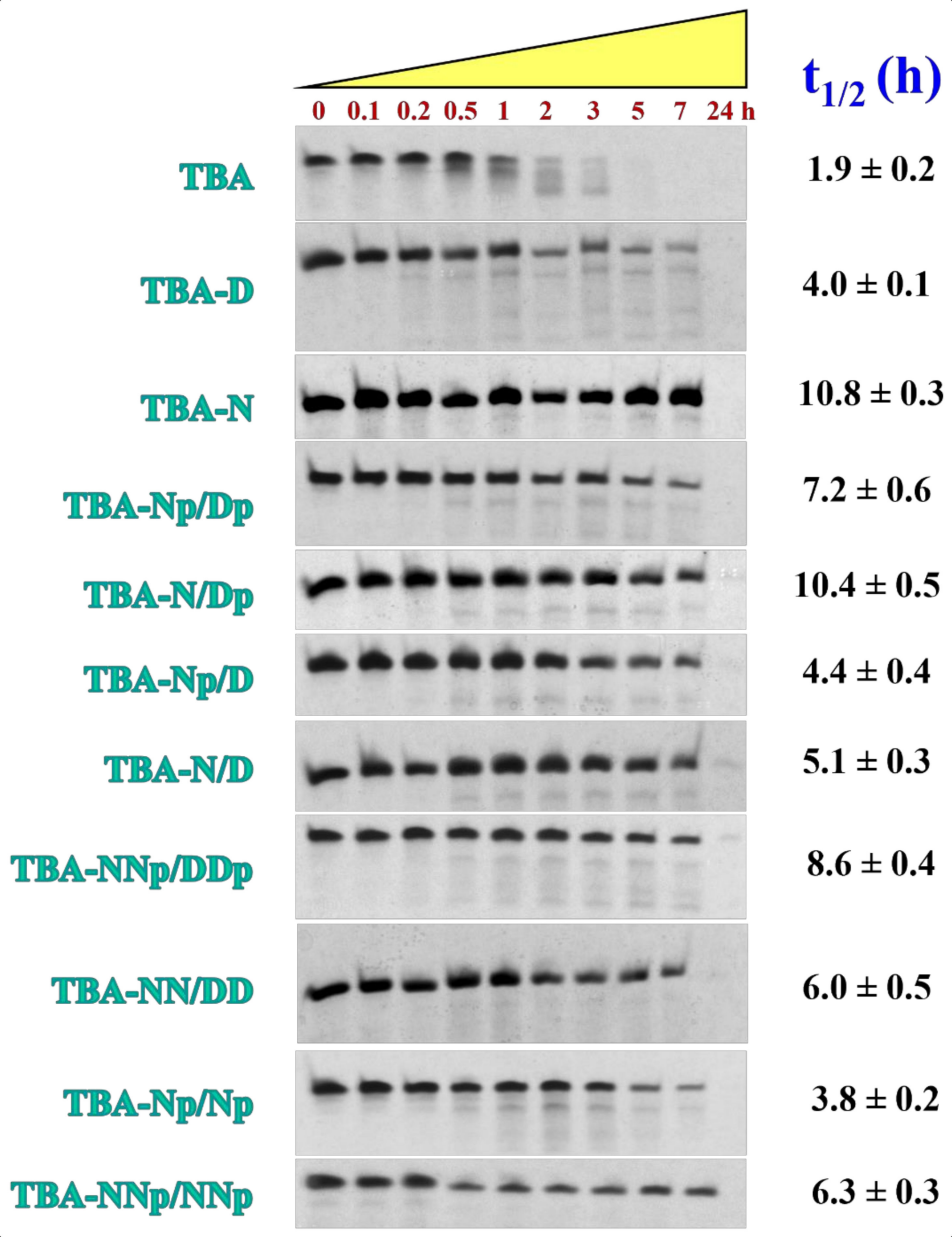
Enzymatic resistance experiments performed on TBA and its derivatives incubated in 80% fetal bovine serum (FBS), as monitored by 20% denaturing polyacrylamide gel electrophoresis for 24 h (time points: 0, 0.1, 0.2, 0.5, 1, 2, 3, 5, 7 and 24 h). For each compound, a representative 20% denaturing PAGE (8 M urea) is reported: samples were loaded at 15 μM, and the gels were run at 200 V constantly, at r.t., for 2.5 h in TBE 1X (running buffer). The intensity of each oligonucleotide band on the gel is also expressed as a percentage of the remaining intact aptamer (see Figure S14) with respect to the untreated oligonucleotide, and the values were fitted with an equation for first order kinetics, allowing the calculation of the half-life in serum of each aptamer (t_1/2_).

The susceptibility of a selected oligonucleotide to nuclease degradation in serum is one of the most crucial parameters for its potential in vivo use. The chemical conjugation at the 5′ and/or 3′ ends of the TBA sequence with a selected probe generally prevents exonuclease attacks. Thus, TBA functionalization with NDI and/or DAN moieties was expected to markedly improve the nuclease resistance of the TBA analogues prepared, in analogy to other end-modified TBA derivatives [32,46,47,90,91,92,93]. In order to evaluate their stability in serum, all the TBA derivatives were tested in their resistance against enzymatic digestion in fetal bovine serum (FBS) in comparison with the unmodified aptamer. To this aim, in parallel experiments, all the aptamers—previously dissolved and annealed in the selected Na^+^-rich buffer (i.e., PBS)—were incubated in 80% (*v*/*v*) FBS at 37 °C and monitored for 24 h. At fixed times, aliquots of these mixtures were collected, supplemented with formamide to immediately stop the nuclease degradation and then loaded on 20% denaturing gel (Figure 6). The intensity of each oligonucleotide band on the gel at the different analyzed time points was then calculated and expressed as a normalized percentage with respect to that of the untreated oligonucleotide. Fitting of the experimental percentages of the remaining aptamer allowed the calculation of the half-life (t_1/2_) values, i.e., the time at which the amount of the sample decayed by 50%. Percentages of each remaining aptamer along with the corresponding fitting curves are represented for each system in Figure S14.

For unmodified TBA, PAGE experiments revealed a progressive reduction of the DNA band intensity up to 3 h and its complete disappearance within 5 h, with a calculated half-life of ca. 1.9 h (Figure 6 and Figure S14). Conversely, after 7 h treatment, all the TBA analogues were still detectable and were completely degraded within 24 h (Figure 6 and Figure S14). Overall, PAGE analysis clearly indicated that all the modified TBAs had from ca. 2 to 6-fold enhanced t_1/2_ values with respect to the unmodified aptamer (Figure 6 and Figure S14, Table S3).

The general trend of serum nuclease resistance—that is, **TBA-N** > **TBA-N/Dp** > **TBA-NNp/DDp** > **TBA-Np/Dp** > **TBA-NNp/NNp** > **TBA-NN/DD** > **TBA-N/D** > **TBA-Np/D** > **TBA-D** > **TBA-Np/Np** > TBA—well correlated with the G4 thermal stability, as determined by CD-derived T_m_ values (Section 2.2.1.2). Indeed, the lowest nuclease stability was found for **TBA-Np/Np** and **TBA-D**—with t_1/2_ values, respectively, of ca. 3.8 and 4.0 h (Figure 6 and Figure S14)—which were the oligonucleotides essentially unfolded at 37 °C in the Na^+^-rich buffer solution (Section 2.2.1.2). On the other hand, the exceptional stability found for **TBA-N** towards nuclease action is definitively in line with its notable G4 thermal stability, as experimentally determined by CD measurements in the Na^+^-containing medium (Section 2.2.1.2).

These data comprehensively confirmed that the higher resistance to enzymatic degradation of the TBA analogues with respect to the unmodified TBA was clearly due to a protective effect of the conjugating groups, making these end-modified oligonucleotides less accessible to the nuclease attack than their linear counterpart, as reported for other TBA derivatives modified at one or both extremities [32,46,47,90,91,92,93]. Along this line, the protection exerted by a propanediol phosphate group is more efficient when it is present at the 3′ end in correlation with the fact that the degradation in serum is mainly controlled by 3′-exonuclease activity [94].

### 2.5. Coagulation Experiments

**Figure 7:**
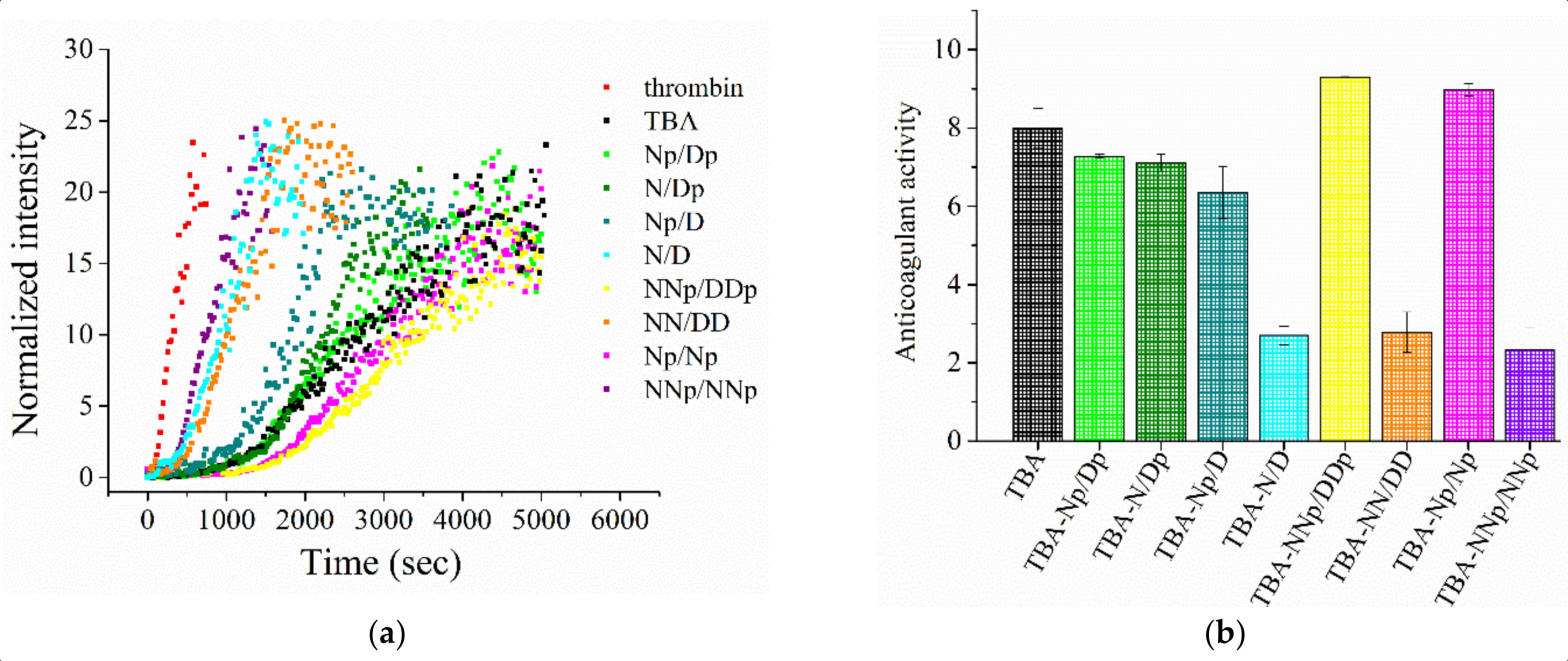
(**a**) Coagulation curves for the pseudo-cyclic TBAs and unmodified TBA at 1:5 thrombin:aptamer molar ratio in PBS, as determined by following the thrombin-catalyzed conversion of fibrinogen into fibrin. A curve in the absence of aptamers is also reported for comparison. (**b**) Anticoagulant activity of pseudo-cyclic TBAs and unmodified TBA. Each experiment was performed at least three times, and the mean value with the standard deviation is reported.

The end-functionalized TBAs were tested for their anticoagulant activity with respect to unmodified TBA by means of light scattering (LS) experiments, by following the thrombin-catalyzed conversion of fibrinogen into fibrin in PBS, used to mimic physiological conditions [46,47,49,50,93]. Normalized LS intensity curves, as a function of time, allow following the fibrinogen–fibrin conversion because the intensity of the scattered light depends on the size of the objects in the solution. Thus, with fibrin being significantly larger than fibrinogen, a marked increase of the scattered light intensity can be detected as soon as fibrin starts forming. Normalized LS intensity curves for all the pseudo-cyclic TBAs at 1:5 thrombin:aptamer molar ratio, and for TBA and in the absence of any oligonucleotide, are reported in Figure 7a.

In the absence of aptamers, a quick and steep increase of intensity is observed, indicating a rapid thrombin-catalyzed conversion of fibrinogen into larger fibrin strands. On the other hand, in the presence of aptamers, less steep curves are recorded, with slopes depending on the aptamer used. In particular, curves recorded in the presence of **TBA-N/D**, **TBA-NN/DD** and **TBA-NNp/NNp** do not differ much from that in the absence of aptamers, indicating that these oligonucleotides scarcely inhibit the thrombin action, while curves obtained in the presence of **TBA-Np/Dp**, **TBA-N/Dp** and **TBA-Np/D** are similar to those in the presence of unmodified TBA, showing a significant thrombin inhibition. Remarkably, curves in the presence of **TBA-NNp/DDp** and **TBA-Np/Np** present the less abrupt rise, an index of a more efficient thrombin inhibition with respect to the native aptamer. With the aim of quantitatively comparing the inhibition properties of pseudo-cyclic TBAs and natural TBA, we calculated the anticoagulant activity of each oligonucleotide as the ratio between the coagulation rate in the absence and presence of the tested oligonucleotides, where the coagulation rates were, in turn, evaluated from the initial slope of the normalized scattered intensities curves.

The anticoagulant activity of pseudo-cyclic TBAs with respect to natural TBA is reported in Figure 7b and Table S3, from which the following trend is derived: **TBA-NNp/DDp** ≅ **TBA-Np/Np** > TBA > **TBA-Np/Dp** ≅ **TBA-N/Dp** > **TBA-Np/D** ≫ **TBA-N/D** ≅ **TBA-NNp/NNp**.

The current results have shown a strong beneficial effect on the inhibition of coagulation activity of charge transfer interactions, associated with the presence of terminal propanediol phosphate groups. Indeed, the TBA analogues lacking these additional groups, i.e., **TBA-N/D** and **TBA-NN/DD**, were poorly active, probably as a consequence of higher rigidity. The poor effect of **TBA-NNp/NNp** was not surprising since it has been shown that its structuration is quite different from the other TBA analogues, hence it was probably not well recognized by thrombin.

## 3. Materials and Methods

### 3.1. General Methods

All the reagents and solvents were of the highest commercially available quality and were used as received. Nuclease-free water, acrylamide/bis-acrylamide (19:1) 40% solution, Tris-Borate-EDTA (TBE) 10×, glycerol formamide and urea were purchased from VWR. Stains-All, ammonium persulfate (APS) and tetramethylethylenediamine (TEMED) were purchased from Sigma Aldrich (Merck Life Science S.r.l., Milan, Italy). Fetal bovine serum (FBS) was provided by Euroclone (Milan, Italy).

Among the oligonucleotides studied, unmodified TBA was purchased from biomers.net GmbH (Ulm/Donau, Germany) as HPLC-purified oligomers. Their identity and purity were proved by MALDI-TOF mass spectrometry and high-performance liquid chromatography (HPLC) data, as provided by the commercial suppliers. All the TBA analogues investigated were synthesized and purified as described below. The purity of all the oligonucleotides was further confirmed by denaturing 20% PAGE analysis.

### 3.2. Synthetic Procedures

1,3-Propanediol-functionalized solid support **1**, dialkoxy naphthalene phosphoramidite **2** and naphthalene diimide phosphoramidite **5** were previously synthesized as reported in the literature [51].

### 3.3. Synthesis of DMTr-NDI-Q-Linker Solid Support 10

To a solution of EDC (1.023 g, 6.59 mmol, 1.4 eq), DMAP (57.5 mg, 0.47 mmol, 0.1 eq) and hydroquinone-*O,O’*-diacetic acid (851 mg, 3.77 mmol, 0.8 eq) in anhydrous pyridine (20 mL), a solution of DMTr-NDI **8** (1.80 g, 4.71 mmol, 1 eq) in anhydrous pyridine (20 mL) was added dropwise. The reaction was stirred for 4 h at 30 °C and monitored by TLC (cyclohexane/AcOEt/NEt_3_ 5:4.5:0.5, *v*/*v*/*v*). After solvent evaporation, the oily residue was dissolved in ethyl acetate (250 mL) and washed three times with water (250 mL). The aqueous layers were extracted with 100 mL of ethyl acetate. The organic layers were combined and dried over Na_2_SO_4_, filtrated and dried under vacuum.

The crude mixture with the mono-Q-linker-NDI **9** (Scheme S1) and a small amount of di-NDI-Q-linker was dissolved in anhydrous pyridine (20 mL) and evaporated to remove traces of water. Then, it was redissolved in 40 mL of pyridine; and EDC (1.023 g, 6.59 mmol), DMAP (57.5 mg, 0.47 mmol) and LCAA-CPG (1.9 g, 1000 Å, 77 μmol of NH_2_/g) were added. The mixture was slowly shaken for 48 h. Then, the CPG support was filtrated, washed with CH_2_Cl_2_ (50 mL) and then dried in desiccator. The resulting DMTr-NDI-Q-CPG was capped with a 1:1 mixture of Cap A (acetic anhydride:pyridine:THF 10:10:80 *v*/*v*/*v*) and Cap B (10% *N*-methylimidazole in THF) for 2 h under slow shaking. Finally, the CPG support was further filtrated, washed with CH_2_Cl_2_ (30 mL) and finally dried in desiccator. A loading of 33 μmol/g was determined with the DMTr assay.

### 3.4. Synthesis of DMTr-DAN-Q-Linker Solid Support 13 

The same protocol used for the DMTr-NDI-Q-CPG was applied for the DMTr-DAN-Q-CPG starting from DMTr-DAN **11** (Scheme S2). A loading of 31 μmol/g was determined with the DMTr assay.

### 3.5. Oligonucleotide Synthesis

Modified aptamers **TBA-Np/Dp TBA-N/Dp**, **TBA-NNp/DDp**, **TBA-Np/Np** and **TAB-NNp/NNp** were synthesized starting from DMTr-Propanediol Q-linker solid support **1**. **TBA-N** was synthesized starting from DMTr-NDI-Q-linker solid support **10**. and **TBA-N/D**, **TBA-Np/D**, **TBA-NN/DD** and **TBA-D** were synthesized starting from DMTr-DAN-Q-linker solid support **13** (Scheme S3). The solid-supported synthesis of oligonucleotides was performed on a 394 ABI DNA synthesizer. All the conventional reagents, solvents for DNA synthesis, *N*2-*tert*-butylphenoxyacetyl-deoxyguanosine and thymidine phosphoramidites were purchased from Link© technologies (Bellshill, UK), ChemGenes© Corporation (Wilmington, MA, USA) and Biosolve© Chimie (Dieuze, France). The aptamers were elongated from **1**, **10** or **13** solid supports on a DNA synthesizer, with a 1.2 to 1.5 μmol scale cycle, according to standard phosphoramidite chemistry protocols. The detritylation step was performed for 65 s using 3% TCA in CH_2_Cl_2_. For the coupling step, benzylmercaptotetrazole (0.3 M in anhydrous CH_3_CN) was used as the activator with modified **2** [51], **5** [51] or **6** phosphoramidites (0.1 M in CH_3_CN, 3 min coupling time) and nucleoside phosphoramidites (0.1 M in CH_3_CN, 90 s coupling time). The capping step was performed with phenoxyacetic anhydride using commercially available solutions (Cap A: phenoxyacetic anhydride:pyridine:THF 10:10:80 *v*/*v*/*v*; Cap B: 10% *N*-methylimidazole in THF) for 20 s. The oxidation step was performed with a standard, diluted iodine solution (0.1 M I_2_, THF:pyridine:water 90:5:5, *v*/*v*/*v*) for 15 s. Oligomers were deprotected and released from the CPG support by treatment with a 7 N solution of NH_3_/MeOH for 4–6 h at 55 °C on a thermoshaker. WARNING: Prolonged treatment with concentrated aqueous ammonia leads to degradation due to the opening of the imides of NDI. The CPG beads were then washed with dry MeOH. The eluate and the washing were pooled and dried on a speed vacuum. Then the beads were washed with water, the eluate was pooled with the previous fractions and dried under vacuum.

### 3.6. Oligomer Treatment and Analysis

For each synthesized oligonucleotide, the residue was dissolved in Milli-Q water and purified by C_18_ reverse-phase HPLC (Macherey-Nagel, Nucleodur 250 × 10 mm, 5 µm) on a Dionex Ultimate 3000 system with an automated injector with a detector UV DAD 3000 at a flow rate of 4.5 mL/min. After this purification step, the oligomers were filtered on Chromafil Xtra H-PTFE-45/25 0.45 µm hydrophilic filter. The amount of the pure TBA analogues was determined by UV-vis analysis at 260 nm and at 90 °C and lyophilized.

MALDI-TOF mass spectra were recorded on Axima© Assurance (Kyoto, Japan) in negative mode using anthranilic acid as matrix. Proper calibration was previously performed with a mixture of oligomers used as reference. TBA analogues were dissolved with 200 µL of water and then 0.75 µL of this solution were mixed with 1 µL of an aqueous solution of anthranilic acid saturated with 50 mM ammonium citrate. This mixture was then applied on a MALDI plate, dried and then analyzed.

### 3.7. Preparation of the Oligonucleotide Samples

Purified and lyophilized oligonucleotides were dissolved in a defined volume of nuclease-free water. Their concentration was determined by UV measurements on a JASCO V-630 UV-vis spectrophotometer equipped with a Peltier Thermostat JASCO ETCS-761, by using a quartz cuvette with a 1 cm path length (1 mL internal volume, Hellma), recording the absorbance at 260 nm and 90 °C. The molar extinction coefficient of 158.480 cm^−1^ M^−1^ was used for both unmodified TBA and its analogues, as calculated for the unstacked oligonucleotides using Oligo Calculator [95]. For the TBA derivatives, it was demonstrated that the inserted DAN and NDI appendages did not provide any relevant contribution to the UV absorbance at 260 nm (Figure 2).

The UV spectra were recorded in the range 220–320 nm with a medium response, a scanning speed of 100 nm/min and a 2.0 nm bandwidth with the appropriate baseline subtracted. Taking a suitable aliquot from the initial stock solutions in H_2_O, all the investigated oligonucleotides were then diluted in the selected K^+^- (10 mM KH_2_PO_4_/K_2_HPO_4_, 70 mM KCl, 0.2 mM EDTA, pH = 7.3) or Na^+^-rich (PBS: 137 mM NaCl, 2.7 mM KCl, 10 mM NaH_2_PO_4_/Na_2_HPO_4_, 1.8 mM KH_2_PO_4_/K_2_HPO_4_, pH = 7.3) phosphate buffer solutions. Thereafter, the samples were annealed by heating the solutions for 5 min at 95 °C and then left to slowly cool to r.t. overnight, so to allow their structuring into the thermodynamically most stable conformations [96]. The annealed samples were eventually kept at 4 °C until their subsequent use.

### 3.8. UV Spectroscopy

**UV-vis spectra.** UV-vis spectra at r.t. were recorded on a JASCO V−750 UV-vis spectrophotometer, by using a quartz cuvette with a 1 cm path length (1 mL internal volume, Hellma). All the investigated oligonucleotides were prepared at 2 μM concentration in HPLC grade water, properly diluting their stock solutions. The absorbance spectra were recorded in the range 220–800 nm using a scanning speed of 200 nm/min and a 2.0 nm bandwidth with the appropriate baseline subtracted. Each experiment was performed in duplicate.

**UV Thermal Difference Spectra.** UV spectra for thermal difference spectra (TDS) analysis were acquired on a JASCO V-630 UV-vis spectrophotometer equipped with a Peltier Thermostat JASCO ETCS-761, by using a quartz cuvette with a 1 cm path length (1 mL internal volume, Hellma). All the investigated oligonucleotides were dissolved in the selected phosphate buffer conditions so to obtain 2 μM solutions, which were then slowly annealed. In detail, the absorbance spectra were recorded at 15 and 90 °C in the range 220–320 nm using a scanning speed of 100 nm/min with the appropriate baseline subtracted. [58,62] Then, TDS data were obtained by subtracting the UV spectrum recorded at a temperature below the T_m_ (15 °C), at which the aptamer is fully structured, from the one obtained at a temperature above the T_m_ (90 °C), where the oligonucleotide G4 structure is fully denatured [61,62,64]. Each experiment was performed in duplicate.

In order to facilitate the comparison of the spectral data, all the obtained differential spectra were then normalized to the maximum of absorbance simply by dividing the raw data in the 220–320 range by its maximum value, so that the highest positive peak gets a Y-value of +1 [61]. From normalized spectra, TDS factors (ΔA_240_/ΔA_295_, ΔA_255_/ΔA_295_ and ΔA_275_/ΔA_295_) were also calculated in both the analyzed saline conditions as the ratios between the absolute absorbance values at different wavelengths [61,63].

**Circular dichroism (CD) spectroscopy.** CD spectra and CD-monitored melting/annealing curves were recorded on a Jasco J-715 spectropolarimeter equipped with a Peltier-type temperature control system (model PTC-348WI), by using a quartz cuvette with a 1 cm path length (3 mL internal volume, Hellma). CD parameters for spectra recording were the following: spectral window 220–320 nm, data pitch 1 nm, band width 2 nm, response 4 s, scanning speed 100 nm/min, 3 accumulations, with the appropriate subtraction of the background scan with proper blank [71]. All the oligonucleotides were analyzed at 2 μM concentration in the selected phosphate buffer solutions.

Thermal denaturation/renaturation curves were recorded following the CD signal—at the maximum ellipticity observed for each oligonucleotide—vs. the temperature (scan rate of 1 °C/min) and recording the spectra from 220 to 320 nm in 5 °C steps, in the 15–90 °C temperature range for both the K^+^- and Na^+^-rich buffer solutions. Each experiment was performed in triplicate.

For both CD spectra and CD-monitored melting/annealing profiles, the molar ellipticity [θ] (deg cm^2^ dmol^−1^) was calculated from the equation [θ] = θ/10 × *l* × *C*, where θ is the observed CD ellipticity in millidegrees, *C* is the oligonucleotide concentration in mol L^−1^, and *l* is the optical path length of the cell in cm.

For the singular value decomposition (SVD) analysis—performed on **TBA-NNp/NNp**—CD spectra were also normalized to molar circular dichroism Δε (M^−1^·cm^−1^) using the equation Δε = θ/(32980 × *C* × *l*). The resulting spectra were then analyzed using the advanced software developed by del Villar-Guerra et al. [72].

Data from thermal denaturation/renaturation curves were also converted into folded fraction (α) calculated as: α = [θ_obs_(T) − θ_U_)/(θ_F_ − θ_U_)], where θ_obs_(T) is the molar ellipticity—at the maximum observed for each oligonucleotide—for each temperature, while θ_F_ and θ_U_ are the molar ellipticity values for the folded (T = 15 °C) and unfolded (T = 90 °C) oligonucleotide, respectively. The T_m_ values were estimated as the maxima of the first derivative plots of the melting/annealing curves and the error associated with the T_m_ determination was ±1 °C. The ΔT_m_, values were calculated by subtracting the measured T_m_ of unmodified TBA from that observed for each TBA analogue.

The CD melting curves showed sigmoidal profiles and were used to determine the thermodynamic change values using the van’t Hoff analysis, as previously performed [50]. In detail, ΔH^0^ and ΔS^0^ values were calculated from the experimental CD profiles by the Marquardt nonlinear least-squares method used by Petersheim and Turner [97] adapted to a monomolecular system [79], under the assumption ΔCp = 0. All the thermodynamic parameters are expressed in kJ/mol as mean values ± SD from the multiple determinations. ΔG^0^ values were calculated at 298 K.

### 3.9. Polyacrylamide Gel Electrophoresis (PAGE) Analysis

**Denaturing PAGE.** Denaturing PAGE experiments were performed according to reported procedures [50,98], with minor modifications. Briefly, 105 pmol of each oligonucleotide sample in water were mixed with formamide (1:2, *v*/*v*) to completely unfold the samples, heated at 95 °C for 5 min, then left in contact with ice until subsequent loading. Thereafter, all the samples—supplemented with 5% glycerol immediately before loading—were analyzed by electrophoresis on 20% denaturing polyacrylamide gels using 8 M urea in TBE 1X as running buffer. The gels were run at r.t., at constant 200 V for 2.5 h, then stained with Stains-All for the night according to the manufacturer’s instructions and finally visualized with a UV transilluminator (BioRad ChemiDoc XRS, Milan, Italy). Each experiment was performed in triplicate.

**Native PAGE.** Slowly, annealed oligonucleotides samples (dissolved at 15 μM) in both the selected K^+^- and Na^+^-rich buffer solutions were loaded on 15% polyacrylamide gels in TBE 1X as running buffer. All the samples were supplemented with 5% glycerol just before loading and then run, under native conditions, at 80 V at r.t. for 2.5 h. Gels were stained with Stains-All in an overnight treatment according to the manufacturer’s instructions and finally visualized with a UV transilluminator (BioRad ChemiDoc XRS, Milan, Italy). Each experiment was performed at least in triplicate.

**Enzymatic stability assays monitored by gel electrophoresis analysis.** The evaluation of the oligonucleotide stability in serum was performed by gel electrophoresis analysis according to reported procedures [50,89], with minor modifications. Briefly, all the oligonucleotides—previously annealed in PBS buffer at 250 μM conc.—were incubated in 80% FBS at 37 °C. Then, at fixed times, 3 μL of the samples (corresponding to 150 pmol) were collected, mixed with formamide (1:2, *v*/*v*) to immediately quench the enzymatic degradation, heated at 95 °C for 5 min, and finally stored at −20 °C until subsequent analysis. Thereafter, all the samples—supplemented with 5% glycerol immediately before loading—were analyzed by electrophoresis on 20% denaturing PAGE using 8 M urea in TBE 1X as running buffer. The gels were run at r.t., at constant 200 V for 2.5 h, then stained with Stains-All in an overnight treatment according to the manufacturer’s instructions and finally visualized with a UV transilluminator (BioRad ChemiDoc XRS, Milan, Italy). Each experiment was performed in triplicate. The intensity of the DNA bands on the gel, at each collected time, was then calculated by using the FiJi software and normalized with respect to the initial one (untreated oligonucleotides). Percentages of the remaining intact oligonucleotide are reported as mean values ± SD for the multiple determinations. Half-life times of each oligonucleotide (t_1/2_) were obtained by fitting the values with an equation for first order kinetics.

**Anticoagulant activity by light scattering measurements**. The anticoagulant activity of the pseudo-cyclic TBA analogues, and of unmodified TBA used as control, was evaluated by means of light scattering (LS) experiments as described before [46,47,49,50,93].

Briefly, a 2.4 μM fibrinogen solution in PBS was placed in a light scattering (LS) cuvette and left to equilibrate in the instrument. After 15 min, an equal volume of a 10 nM thrombin solution was added in order to obtain a final concentration of 1.2 μM for fibrinogen and 5 nM for thrombin. Immediately after the thrombin addition, the light scattered intensity was registered every 2 s by using a home-made instrument composed of a Photocor compact goniometer, a SMD 6000 Laser Quantum 50 mW light source operating at 5325 Å, a photomultiplier (PMT−120-OP/B) and a correlator (Flex02−01D) from Correlator.com. The intensity was measured at scattering angle of 90° and at (25.00 ± 0.05) °C, with temperature controlled through the use of a thermostat bath. When the aptamers were tested, they were added to fibrinogen solutions so to have a final thrombin:aptamer molar ratio of 1:5. All the experiments were performed at least in triplicate.

Coagulation curves were obtained by plotting the normalized scattered intensity *nI*

$$nI=\frac{I-I_{0}}{I_{0}}$$

where I is the scattering intensity at time *t* and $I_{0}$ the scattering intensity at the start of the experiment, as a function of time.

The coagulation rate in the absence and in the presence of aptamers was evaluated by linearly fitting the initial increase of nI upon the lag time, while the anticoagulant activity was calculated as the ratio between the coagulation rate in the presence of thrombin alone and that in the presence of both thrombin and each aptamer.

## 4. Conclusions

The 15-mer 5′-GGT TGG TGT GGT TGG-3′ showed significant antithrombin activity; however, low resistance to nucleases and poor bioavailability prevented its use as a suitable anticoagulant drug. The design of optimized analogues of this aptamer is a very challenging task, requiring a critical compromise between nuclease resistance, lipophilicity, thrombin recognition and anticoagulation activity. To improve the enzymatic resistance and thermal stability of the TBA G-quadruplex, a general and widely exploited approach is based on conjugation with proper probes inserted as end-modifications to the TBA oligonucleotide sequence. This approach allows maintaining the essential core of the aptamer and avoiding detrimental interference with the conjugating groups of the G4 structure, thereby maintaining the ability to recognize the thrombin binding site, i.e., exosite I [32]. All the pseudo-cyclic TBA analogues investigated proved to be up to ca. 6-fold more resistant to nucleases than natural TBA. Likewise, all the TBA analogues with one or two DAN/NDI pairs exhibited higher T_m_ values of up to 15 and 17 °C, in both the Na^+^- and K^+^-rich buffers, respectively, in comparison with the natural TBA, thanks to their cyclic-like structure realized by charge transfer interactions between the conjugating groups. The introduction of aromatic moieties, inducing higher lipophilicity—even when partly compensated by the presence of propanediol monophosphate groups—should increase the aptamer’s bioavailability. Interestingly, as reflected by their higher retention times in reverse phase HPLC compared to native TBA (Figure S15), all the TBA analogues exhibited higher lipophilicity, suggesting that—once in vivo—they could be less rapidly cleared from the body than TBA.

As also previously observed, the thermal stability of the G4 structures in the TBA analogues is not strictly correlated with their anticoagulation activity levels [46,47]. Indeed, in the investigated series, **TBA-NNp/DDp** and **TBA-Np/Np** showed the highest anticoagulation activity levels, exhibiting T_m_ values of 44 and 34 °C, respectively, in the Na^+^-rich buffer. The most thermally stable **TBA-N/D**, on the other hand, with a T_m_ of 51 °C, proved to be the worst inhibitor. This trend could be explained by better accommodation of **TBA-NNp/DDp** and **TBA-Np/Np** in thrombin exosite I, resulting in improved binding to the protein and better anticoagulating activity. From a structural point of view, for these optimized TBA analogues, the attractive stacking interactions of the terminal groups can be partially counterbalanced by the repulsive effects of the end propanediol phosphates, and thus lead to more flexible and thus more adaptive, even though less stable, G-quadruplex structures than that of the highly stabilized **TBA-N/D**.

Among the pseudo-cyclic TBA analogues stabilized by charge transfer interactions of the DAN and NDI moieties at the ends of the aptamer, **TBA-NNp/DDp** appeared to be the best candidate of the set we studied. Indeed, it exhibited a more stable G-quadruplex structure (ΔT_m_ of + 8 °C in the selected Na^+^ rich buffer), a 4.5-fold increase of nuclease resistance, higher lipophilicity and better anticoagulation activity (1.2-fold) than natural TBA (Table S3).

Compared to the covalently linked cyclic TBAs we previously synthesized [46,47], the here-described TBA variants displayed, as a whole, from good to high G-quadruplex stabilization and anti-thrombin activity. These findings support our hypothesis on the benefit of charge transfer or π–π stacking interactions, which allowed us producing pseudo-cyclic TBA analogues, as an alternative to covalently linked cyclic analogues. In this design, one can maintain sufficient flexibility in the aptamers for their optimal accommodation in the thrombin binding site and thus exert an improved anticoagulating effect.

## Figures and Tables

**Table 1:** Apparent melting temperature values obtained by CD-monitored thermal experiments for heating and cooling profiles of the studied oligonucleotides in the selected K^+^-rich and Na^+^-rich buffer solutions. Apparent T_m_ values were estimated from the maxima of the first derivative plots. Each T_m_ value was calculated as the average of three independent measurements. The error associated with the T_m_ determination was ±1 °C. ΔT_m_ was calculated by subtracting the measured T_m_ of unmodified TBA from that observed for each TBA analogue. * Followed at 268 nm.

Aptamer	CD-Melting and Annealing Experiments					
	K^+^-Rich Buffer			Na^+^-Rich Buffer		
	Melting T_m_ (°C)	ΔT_m_ (°C)	Annealing T_m_ (°C)	Melting T_m_ (°C)	ΔT_m_ (°C)	Annealing T_m_ (°C)
**TBA**	51	0	49	36	-	35
**TBA-D**	45	−6	43	31	−5	30
**TBA-N**	64	+13	62	51	+15	49
**TBA-Np/Dp**	51	0	51	38	+2	37
**TBA-N/Dp**	62	+11	61	48	+12	48
**TBA-Np/D**	57	+6	56	42	+6	42
**TBA-N/D**	68	+17	66	51	+15	49
**TBA-NNp/DDp**	62	+11	61	44	+8	43
**TBA-NN/DD**	61	+10	60	42	+6	40
**TBA-Np/Np**	49	−2	48	34	−2	33
**TBA-NNp/NNp** *	56	+5	55	44	+8	43

## Data Availability

Not applicable.

## Supplementary Materials

The Supplementary Materials are available online at https://www.mdpi.com/article/10.3390/ijms22179510/s1.
