# Supplementary material for: Charge-Transfer Interactions Stabilize G-Quadruplex-Forming Thrombin Binding Aptamers and Can Improve Their Anticoagulant Activity
Source: Int J Mol Sci. 2021 Sep 2;22(17):9510. doi: 10.3390/ijms22179510 (PMC8430690; doi:10.3390/ijms22179510)
Supplement: Supplementary file 1 [file ijms-22-09510-s001.zip › ijms-1339059-supplementary.pdf]

Supporting Information

to the manuscript:

Charge-transfer interactions stabilize G-quadruplex-forming thrombin binding aptamers and can improve their anticoagulating activity

Kévan Pérez de Carvasal,§[a] Claudia Riccardi,§[b] Irene Russo Krauss,[b,c] Domenico Cavasso,[b,c] Luigi Paduano,[b,c] Jean-Jacques Vasseur,[a] Michael Smietana,[a] François Morvan,[a,*] Daniela Montesarchio[b,*]

[a]*Institut des Biomolécules Max Mousseron, Univ. Montpellier, CNRS, ENSCM, Montpellier, France*
[b]*Department of Chemical Sciences, University of Naples Federico II, Via Cintia 21, I-80126, Naples, Italy*
[c]*CSGI – Consorzio Interuniversitario per lo Sviluppo dei Sistemi a Grande Interfase, Via della Lastruccia 3, I-50019, Sesto Fiorentino (Fi), Italy*

§These authors contributed equally to this work.

*Corresponding authors. E-mail addresses:
francois.morvan@umontpellier.fr (F. Morvan); daniela.montesarchio@unina.it (D. Montesarchio)

Table of contents

Scheme S1. Synthesis of DMTr-NDI-Q-linker solid support 10. 3
Scheme S2. Synthesis of DMTr-DAN-Q-linker solid support 13. 3
Scheme S3. Synthesis of the TBA analogues 4
Figure S1. 20% denaturing polyacrylamide gel electrophoresis analysis 5
Table S1. TDS factor analysis 6
Figure S2. Prediction of G4 topologies adopted by TBA-NNp/NNp by SVD analysis 7
Figure S3. CD analysis of TBA 8
Figure S4. CD analysis of TBA-D 9
Figure S5. CD analysis of TBA-N 100
Figure S6. CD analysis of TBA-Np/Dp 111
Figure S7. CD analysis of TBA-N/Dp 122
Figure S8. CD analysis of TBA-Np/D 133
Figure S9. CD analysis of TBA-N/D 144
Figure S10. CD analysis of TBA-NNp/DDp 15
Figure S11. CD analysis of TBA-NN/DD 166
Figure S12. CD analysis of TBA-Np/Np 177
Figure S13. CD analysis of TBA-NNp/NNp 188
Table S2. Standard thermodynamic parameters from van't Hoff analysis 19
Figure S14. Nuclease resistance experiments on the TBA analogues 200
Figure S15. Comparative reverse phase C18 HPLC analysis of the TBA analogues 211
Table S3. Summary of aptamers properties. 222
Table S4. Reverse phase C18 HPLC and MALDI-TOF MS analysis of the TBA analogues 22

References 322

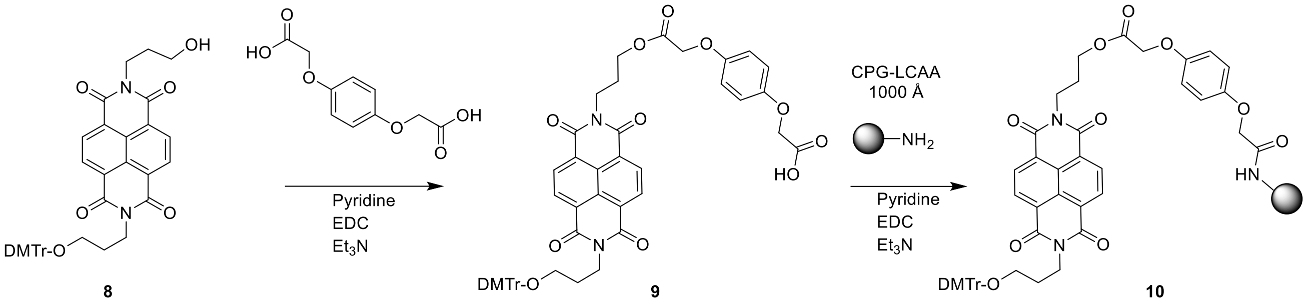

Scheme S1. Synthesis of DMTr-NDI-Q-linker solid support 10.

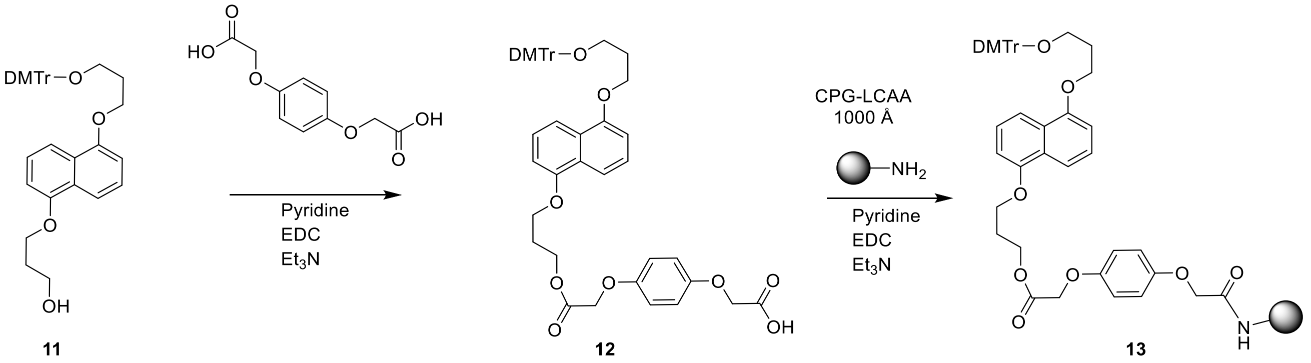

Scheme S2. Synthesis of DMTr-DAN-Q-linker solid support 13.

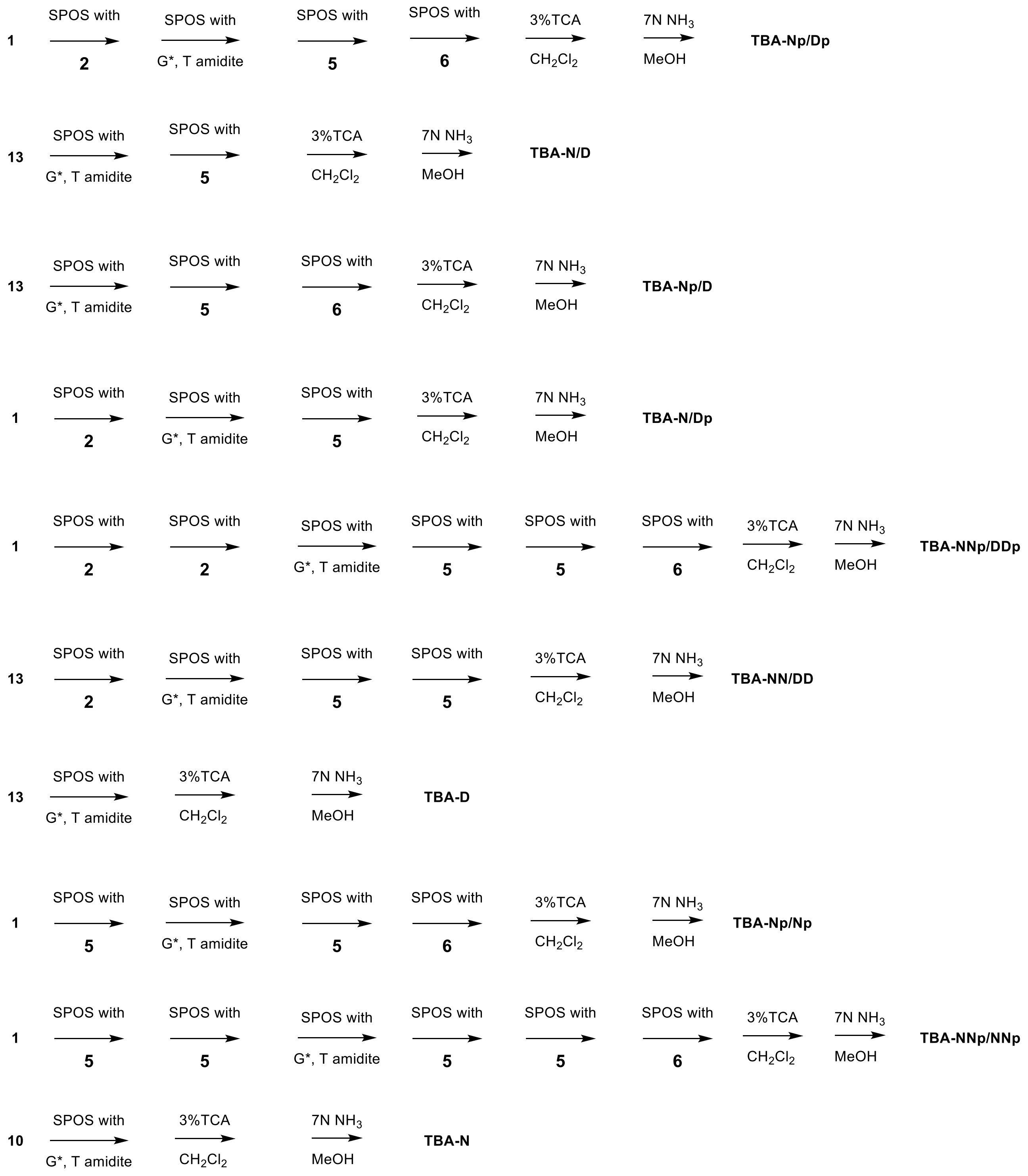

Scheme S3. Synthesis of the TBA analogues.

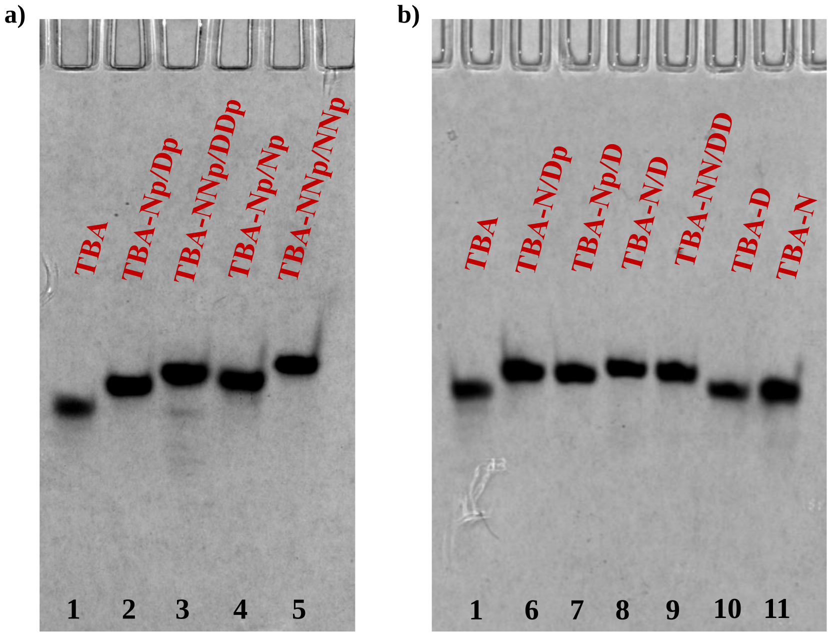

Figure S1. 20% denaturing polyacrylamide gel electrophoresis analysis. Representative 20% denaturing polyacrylamide gel electrophoresis (8 M urea) at 10.5 μM sample concentration, run at constant 200 V at r.t. for 2.5 h in TBE 1X as running buffer. (**a**) Lane 1: TBA; lane 2: **TBA-Np/Dp**; lane 3: **TBA-NNp/DDp**; lane 4: **TBA-Np/Np**; lane 5: **TBA-NNp/NNp**; (**b**) Lane 1: TBA; lane 6: **TBA-N/Dp**; lane 7: **TBA-Np/D**; lane 8: **TBA-N/D**; lane 9: **TBA-NN/DD**; lane 10: **TBA-D**; lane 11: **TBA-N**.

Table 1. TDS factor analysis.

Aptamer	TDS factors					
	K^+-rich buffer			Na^+-rich buffer		
	$\Delta A_{240}/\Delta A_{295}$	$\Delta A_{255}/\Delta A_{295}$	$\Delta A_{275}/\Delta A_{295}$	$\Delta A_{240}/\Delta A_{295}$	$\Delta A_{255}/\Delta A_{295}$	$\Delta A_{275}/\Delta A_{295}$
TBA	0.7	0.1	0.6	0.7	0.1	0.8
TBA-D	0.4	0.3	0.1	0.8	0.1	0.8
TBA-N	0.7	0.2	0.4	0.6	0.3	0.4
TBA-Np/Dp	1.5	0.3	0.4	0.7	0.1	0.9
TBA-N/Dp	1.1	0.6	0.3	1.4	0.4	0.2
TBA-Np/D	1.5	0.3	0.6	1.2	0.2	0.2
TBA-N/D	1.8	0.1	0.5	1.6	0.2	0.3
TBA-NNp/DDp	1.9	0.3	0.6	0.6	0.4	0.4
TBA-NN/DD	1.1	0.2	0.5	1.3	0.1	0.7
TBA-Np/Np	1.0	0.2	0.3	0.7	0.1	0.7
TBA-NNp/NNp	1.4	0.1	0.6	0.8	0.2	0.7

Ratios between values at different absorbance wavelengths as calculated from normalized TDS spectra for the indicated oligonucleotides in both the selected phosphate buffer solutions, according to literature protocols [1].

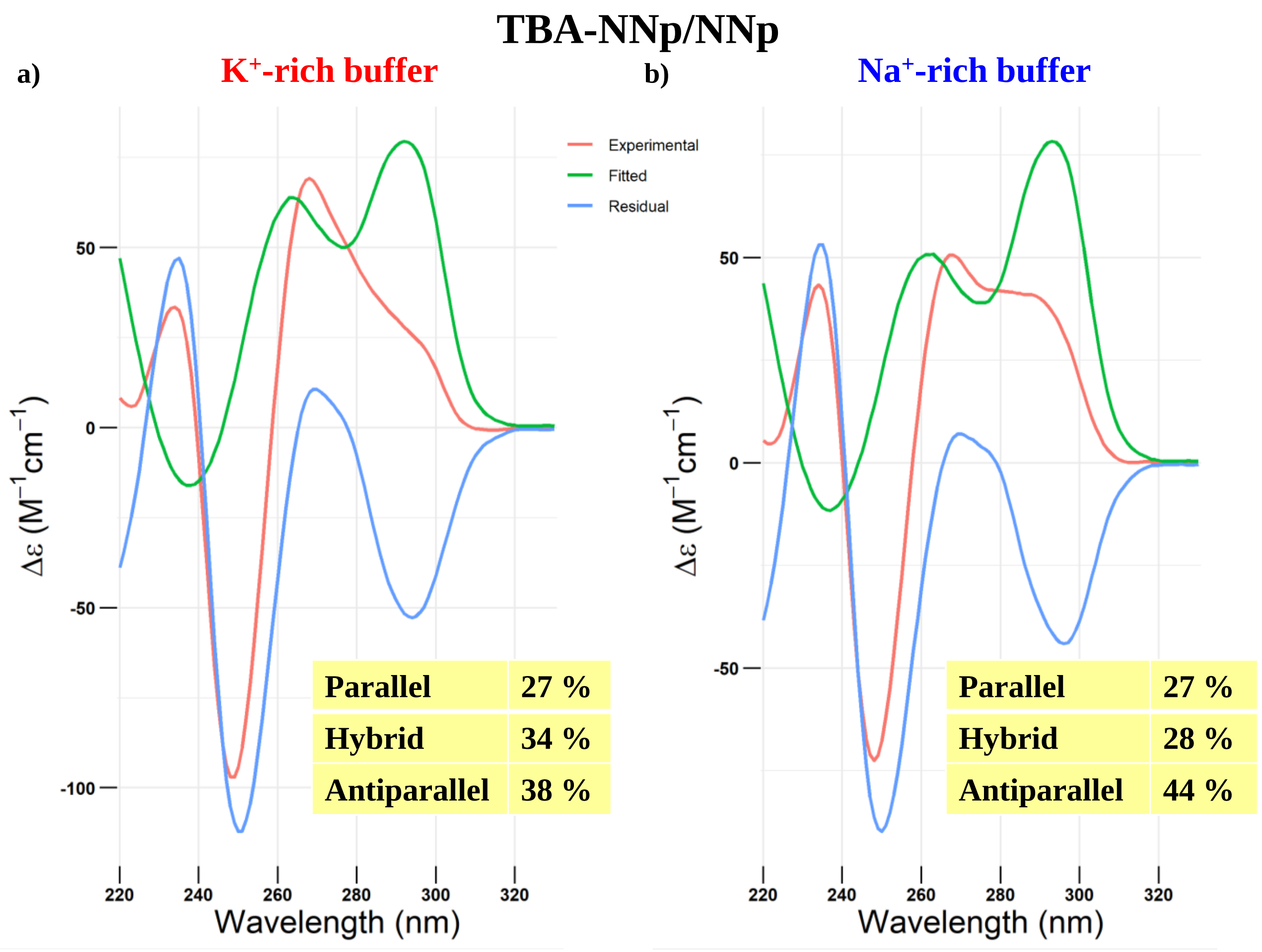

Figure S2. Prediction of G4 topologies adopted by TBA-NNp/NNp by SVD analysis. Prediction of the relative abundance of the G4 topologies adopted by **TBA-NNp/NNp**, obtained by singular value decomposition (SVD) analysis of the CD spectra recorded in both the selected buffer solutions, performed by exploiting the software developed by del Villar-Guerra *et al.*[2] Deviations from 100% (± 1%) are due to significant digits approximation of the values originally obtained by the simulations.

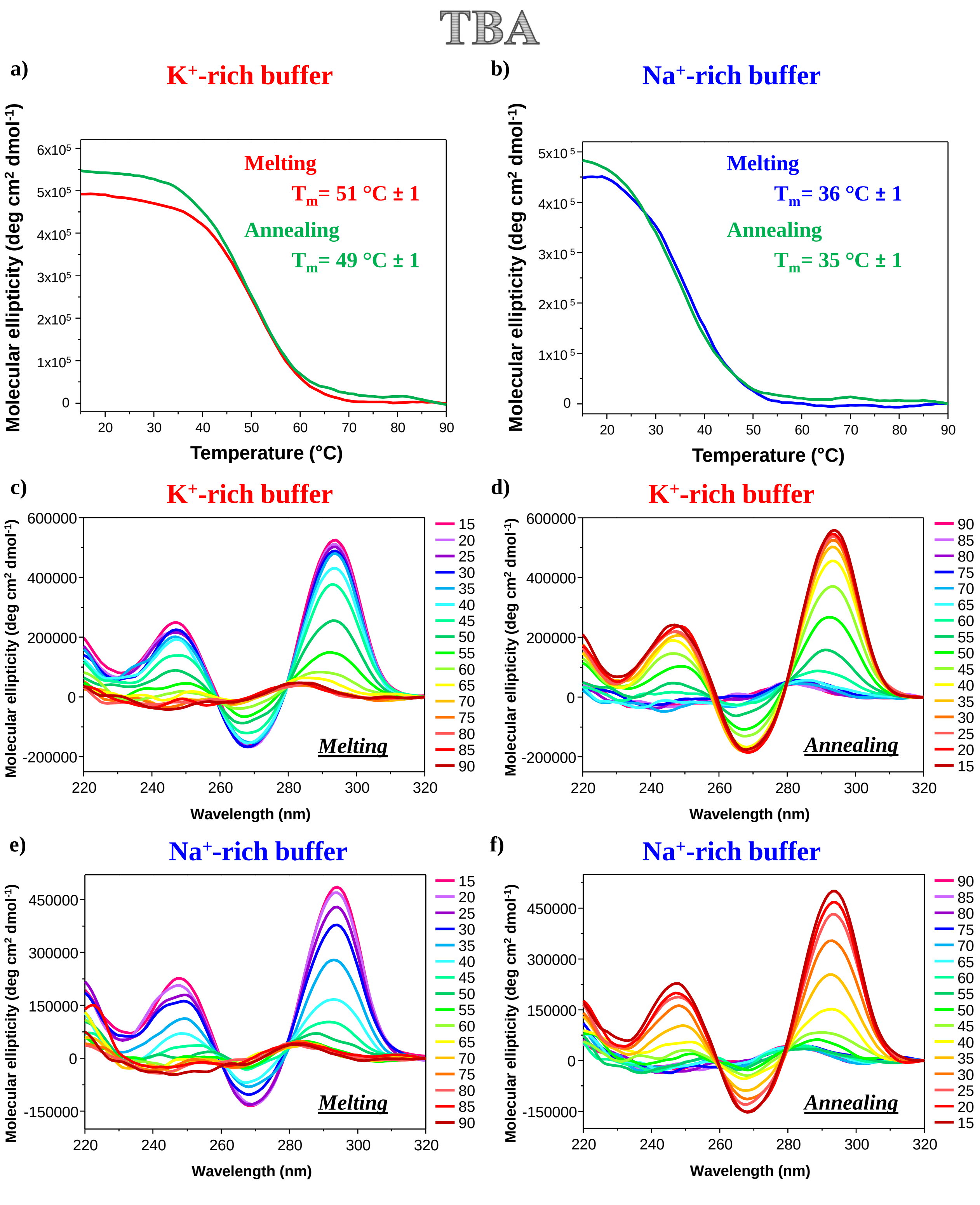

Figure S3. CD analysis of TBA. CD-melting and CD-annealing profiles (**a**, **b**) of TBA at 2 μM concentration in both the selected K^+- (**a**) and Na^+-rich (**b**) buffer solutions. The CD curves were obtained following the CD signal at 294 nm in both saline conditions, with a temperature scan rate of 1 °C/min. The overlapped CD spectra of TBA were recorded every 5 °C during the melting (**c**, **e**) and cooling processes (**d**, **f**) in both the selected K^+- (**c**, **d**) and Na^+-rich (**e**, **f**) buffer solutions.

TBA-D

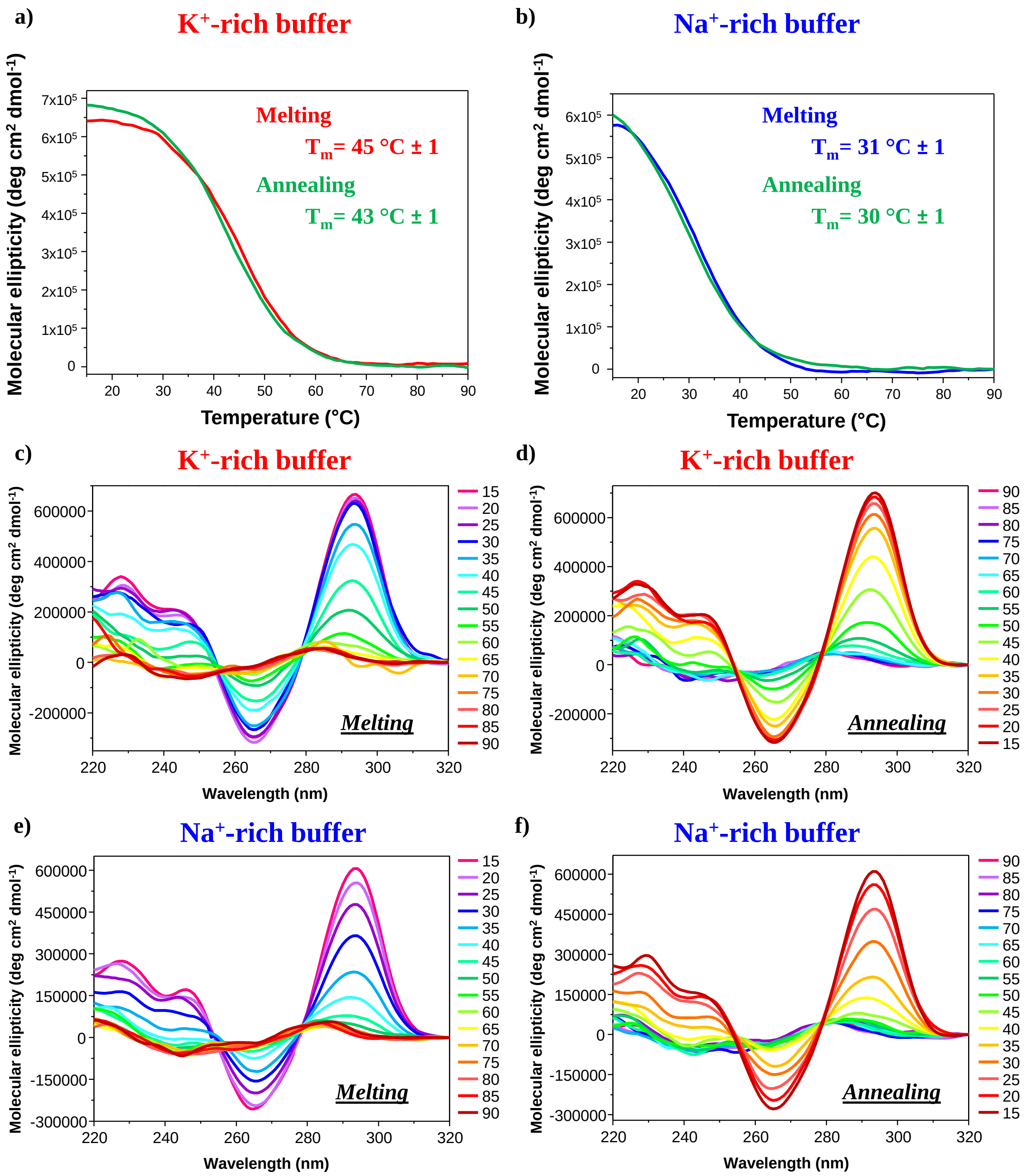

Figure S4. CD analysis of TBA-D. CD-melting and CD-annealing profiles (**a**, **b**) of **TBA-D** at 2 μM concentration in both the selected K^+- (**a**) and Na^+-rich (**b**) buffer solutions. The CD curves were obtained following the CD signal at 294 nm in both saline conditions, with a temperature scan rate of 1 °C/min. Overlapped CD spectra of **TBA-D** were recorded every 5 °C during the melting (**c**, **e**) and cooling processes (**d**, **f**) in both the selected K^+- (**c**, **d**) and Na^+-rich (**e**, **f**) buffer solutions.

TBA-N

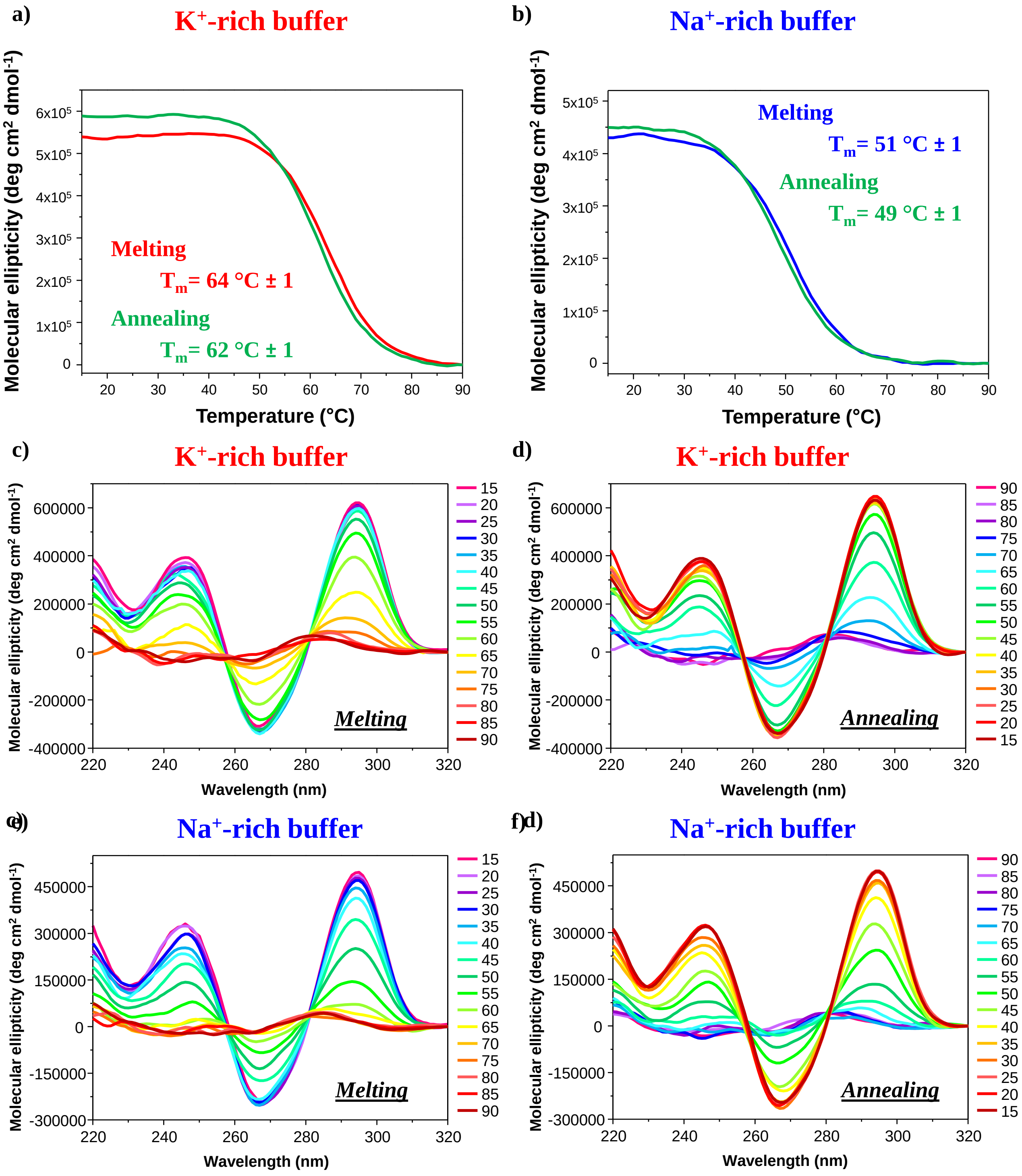

Figure S5. CD analysis of TBA-N. CD-melting and CD-annealing profiles (**a**, **b**) of **TBA-N** at 2 μM concentration in both the selected K^+- (**a**) and Na^+-rich (**b**) buffer solutions. The CD curves were robtained following the CD signal at 294 nm in both saline conditions, with a temperature scan rate of 1 °C/min. The overlapped CD spectra of **TBA-N** were recorded every 5 °C during the melting (**c**, **e**) and cooling processes (**d**, **f**) in both the selected K^+- (**c**, **d**) and Na^+-rich (**e**, **f**) buffer solutions.

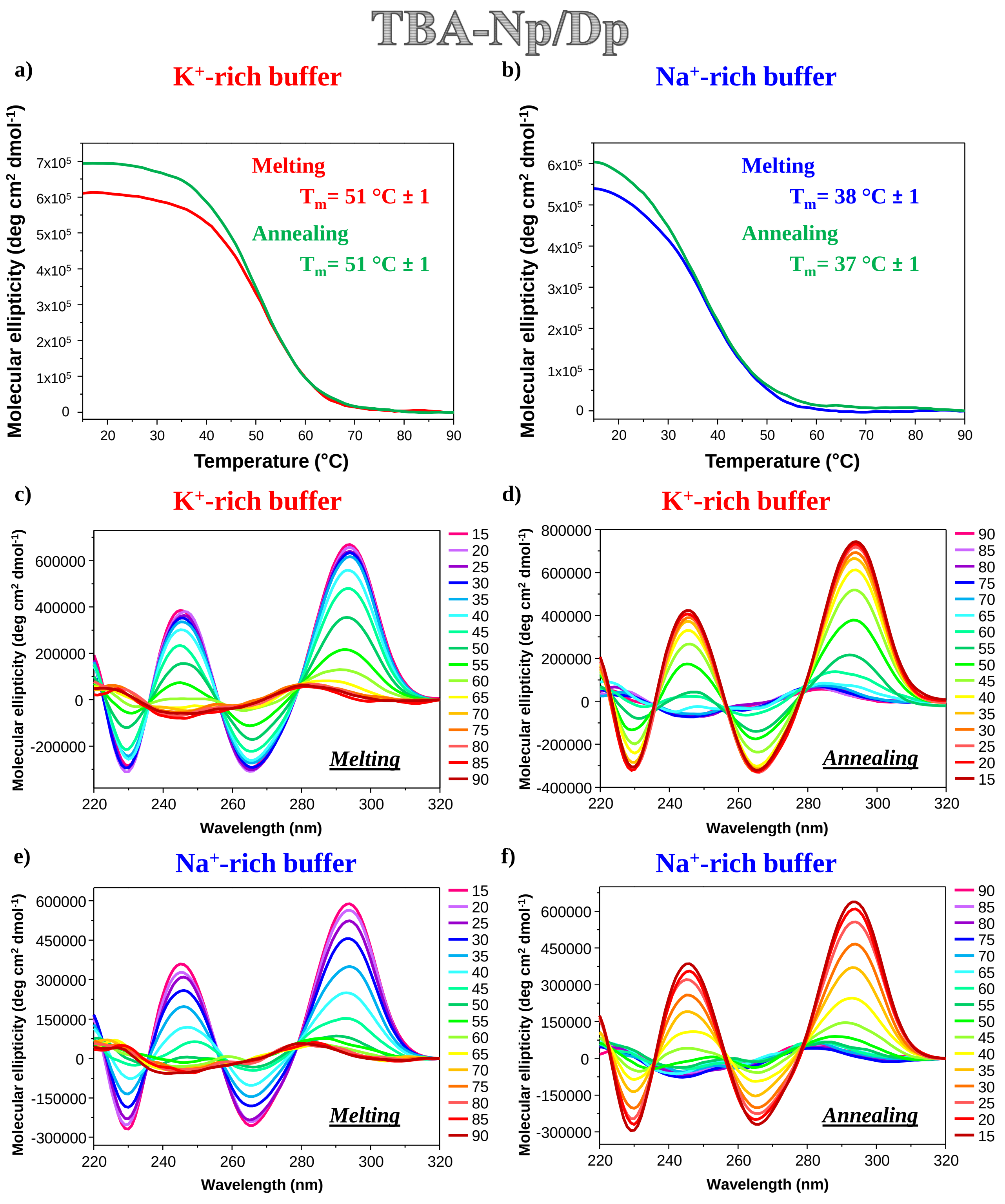

Figure S6. CD analysis of TBA-Np/Dp. CD-melting and CD-annealing profiles (**a**, **b**) of **TBA-Np/Dp** at 2 μM concentration in both the selected K^+- (**a**) and Na^+-rich (**b**) buffer solutions. The CD curves were obtained following the CD signal at 294 nm in both saline conditions, with a temperature scan rate of 1 °C/min. The overlapped CD spectra of **TBA-Np/Dp** were recorded every 5 °C during the melting (**c**, **e**) and cooling processes (**d**, **f**) in both the selected K^+- (**c**, **d**) and Na^+-rich (**e**, **f**) buffer solutions.

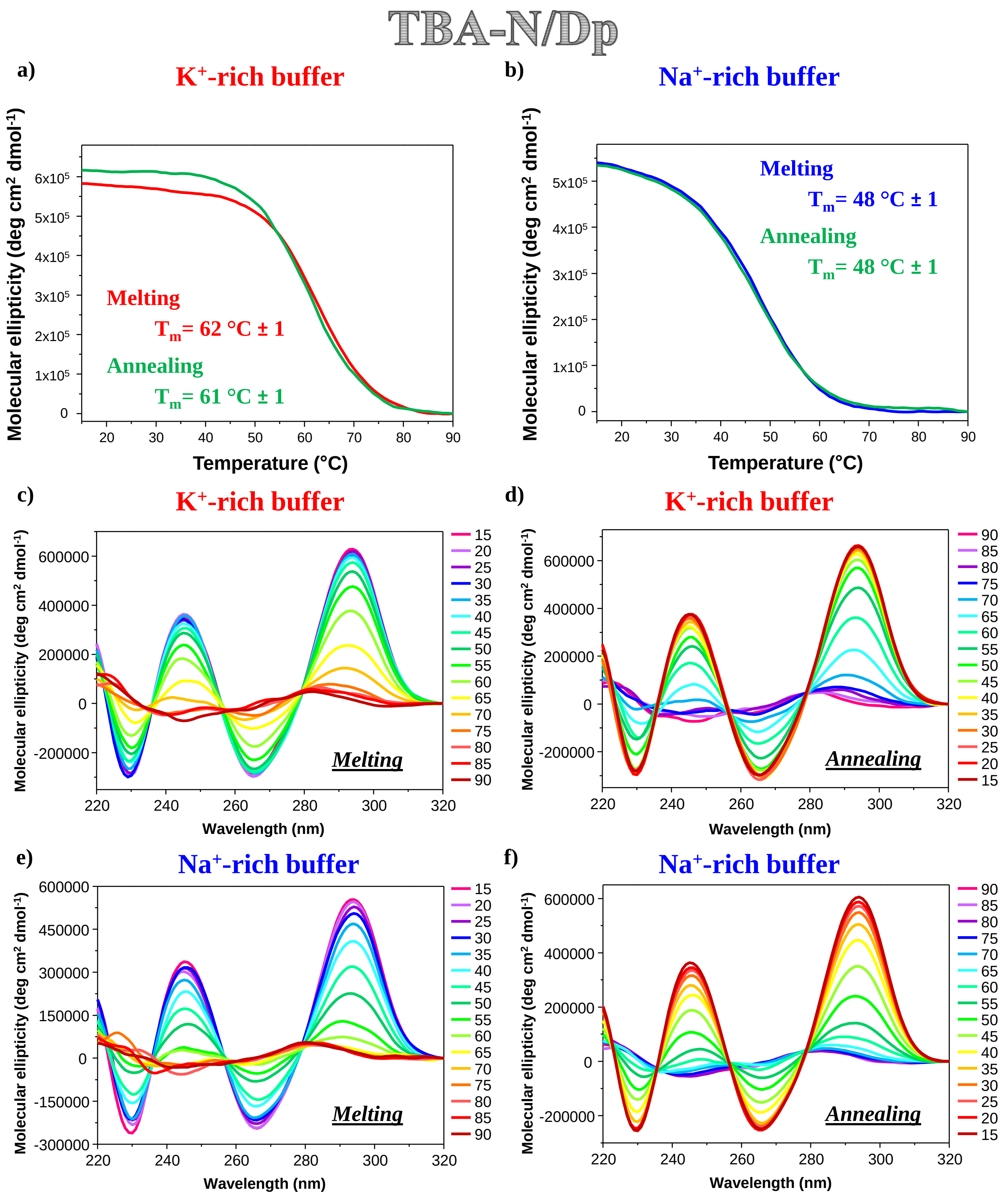

Figure S7. CD analysis of TBA-N/Dp. CD-melting and CD-annealing profiles (**a**, **b**) of **TBA-N/Dp** at 2 μM concentration in both the selected K^+- (**a**) and Na^+-rich (**b**) buffer solutions. The CD curves were obtained following the CD signal at 294 nm in both saline conditions, with a temperature scan rate of 1 °C/min. The overlapped CD spectra of **TBA-N/Dp** were recorded every 5 °C during the melting (**c**, **e**) and cooling processes (**d**, **f**) in both the selected K^+- (**c**, **d**) and Na^+-rich (**e**, **f**) buffer solutions.

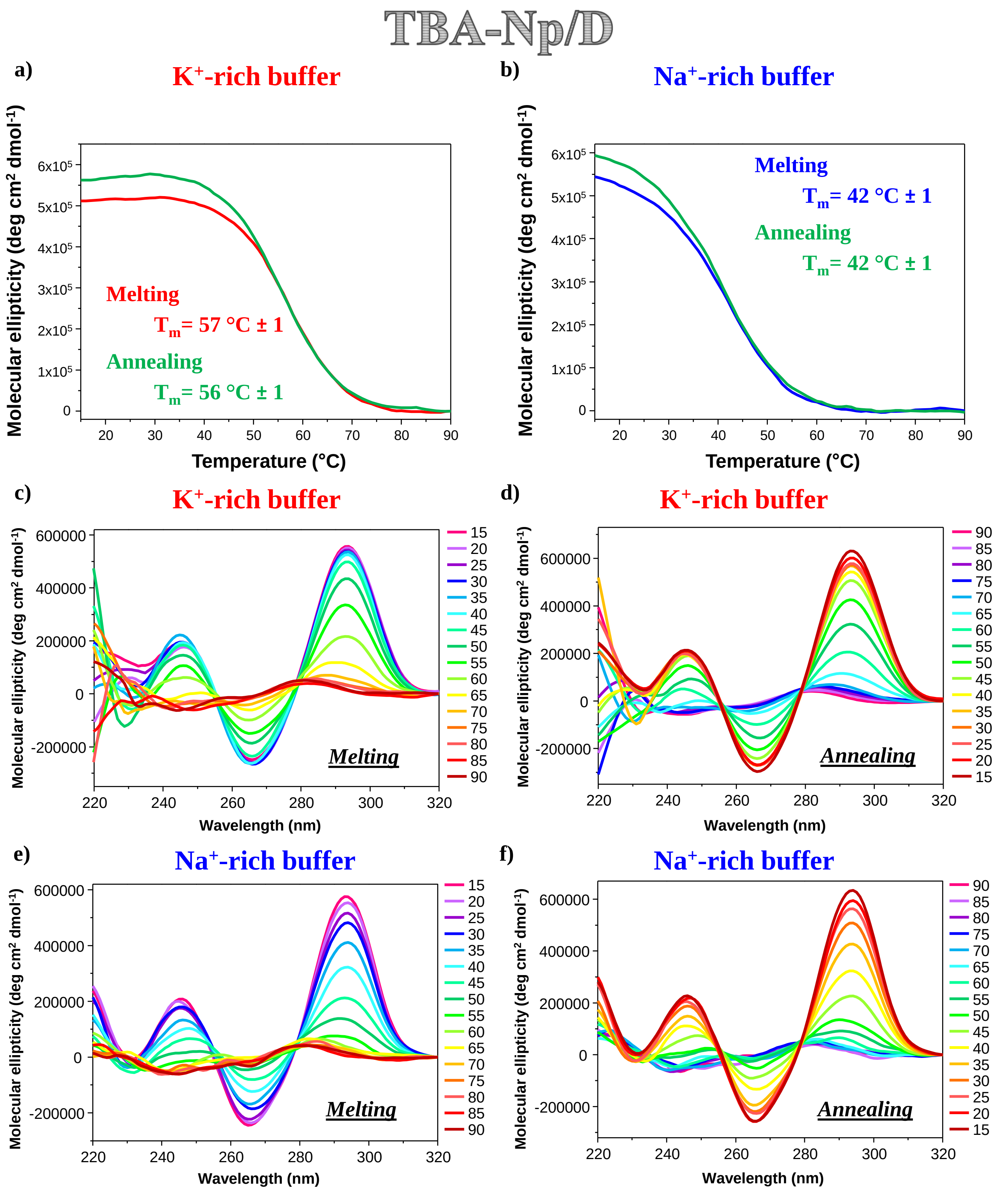

Figure S8. CD analysis of TBA-Np/D. CD-melting and CD-annealing profiles (**a**, **b**) of **TBA-Np/D** at 2 μM concentration in both the selected K^+- (**a**) and Na^+-rich (**b**) buffer solutions. The CD curves were obtained following the CD signal at 294 nm in both saline conditions, with a temperature scan rate of 1 °C/min. The overlapped CD spectra of **TBA-Np/D** were recorded every 5 °C during the melting (**c**, **e**) and cooling processes (**d**, **f**) in both the selected K^+- (**c**, **d**) and Na^+-rich (**e**, **f**) buffer solutions.

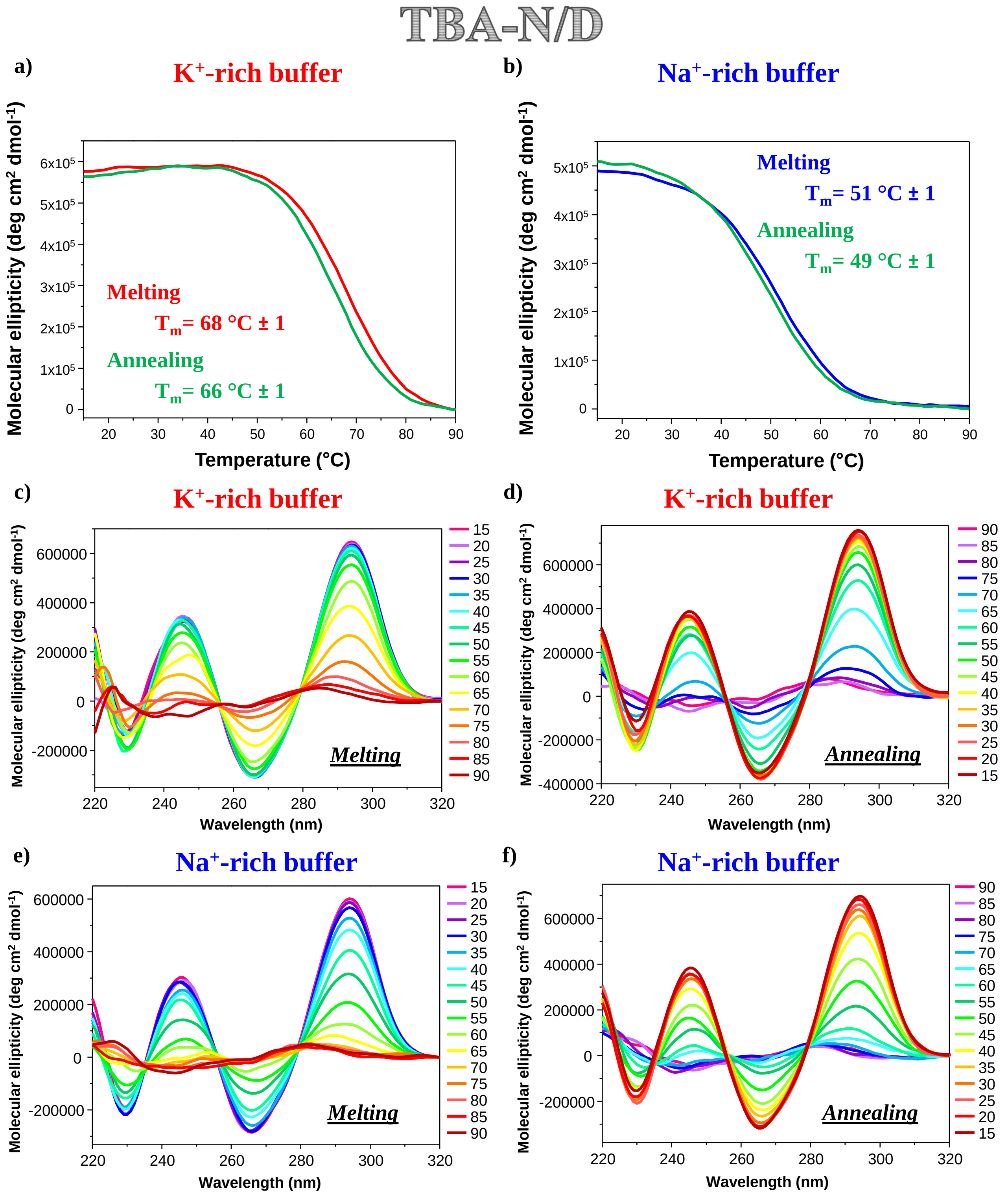

Figure S9. CD analysis of TBA-N/D. CD-melting and CD-annealing profiles (**a**, **b**) of **TBA-N/D** at 2 μM concentration in both the selected K^+- (**a**) and Na^+-rich (**b**) buffer solutions. The CD curves were obtained following the CD signal at 294 nm in both saline conditions, with a temperature scan rate of 1 °C/min. The overlapped CD spectra of **TBA-N/D** were recorded every 5 °C during the melting (**c**, **e**) and cooling processes (**d**, **f**) in both the selected K^+- (**c**, **d**) and Na^+-rich (**e**, **f**) buffer solutions.

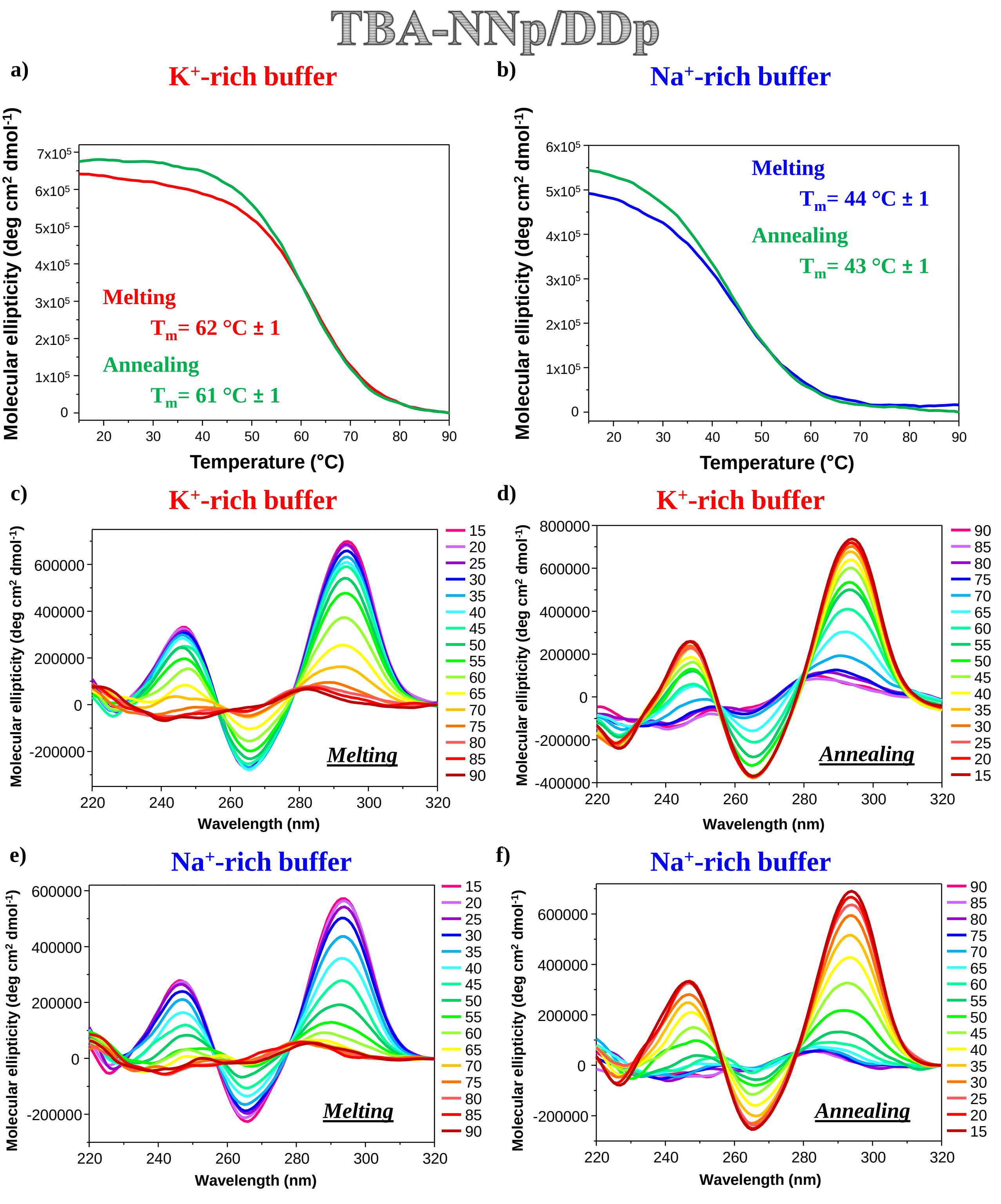

Figure S10. CD analysis of TBA-NNp/DDp. CD-melting and CD-annealing profiles (**a**, **b**) of **TBA-NNp/DDp** at 2 μM concentration in both the selected K^+- (**a**) and Na^+-rich (**b**) buffer solutions. The CD curves were obtained following the CD signal at 294 nm in both saline conditions, with a temperature scan rate of 1 °C/min. The overlapped CD spectra of **TBA-NNp/DDp** were recorded every 5 °C during the melting (**c**, **e**) and cooling processes (**d**, **f**) in both the selected K^+- (**c**, **d**) and Na^+-rich (**e**, **f**) buffer solutions.

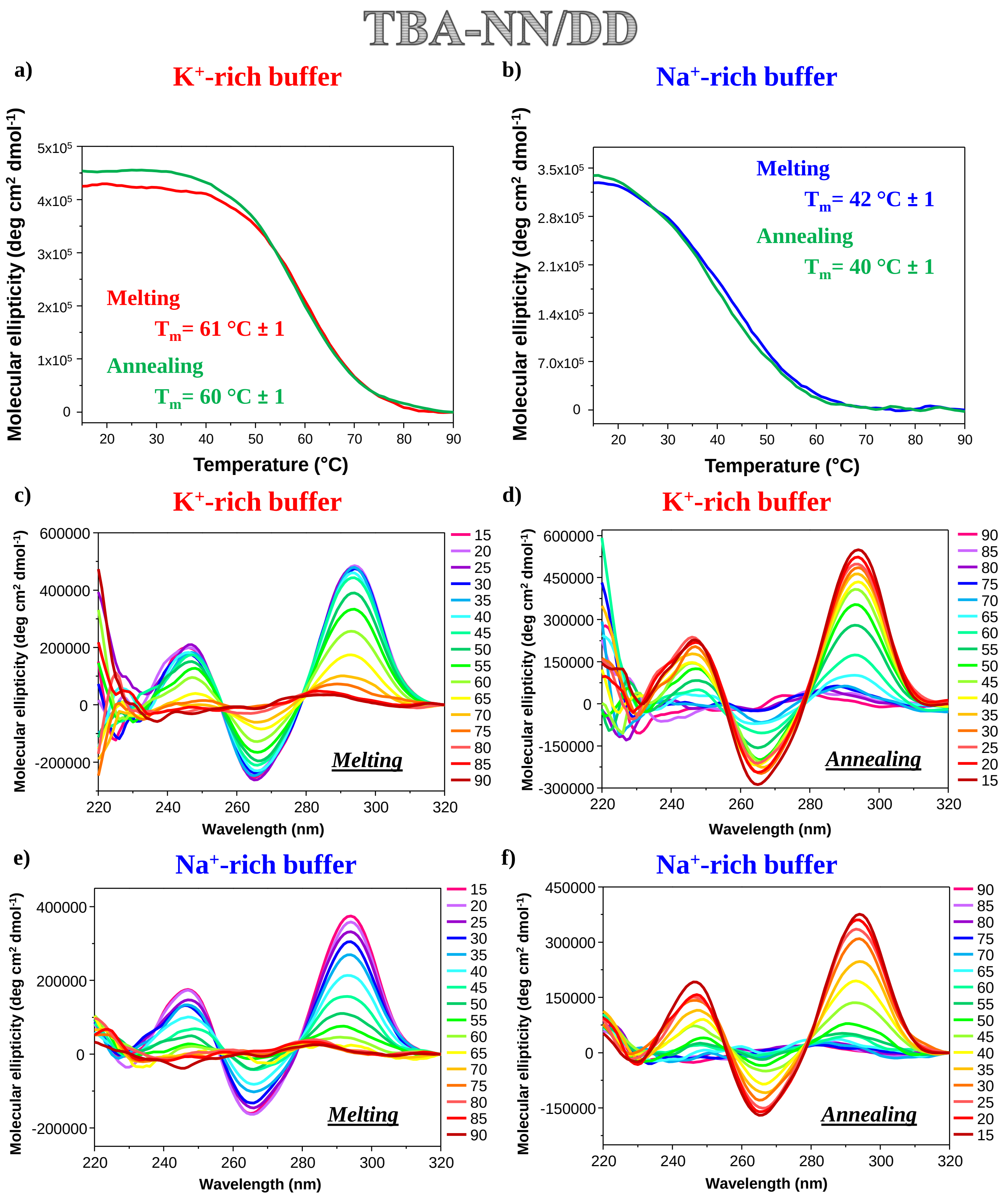

Figure S11. CD analysis of TBA-NN/DD. CD-melting and CD-annealing profiles (**a**, **b**) of **TBA-NN/DD** at 2 μM concentration in both the selected K^+- (**a**) and Na^+-rich (**b**) buffer solutions. The CD curves were obtained following the CD signal at 294 nm in both saline conditions, with a temperature scan rate of 1 °C/min. The overlapped CD spectra of **TBA-NN/DD** were recorded every 5 °C during the melting (**c**, **e**) and cooling processes (**d**, **f**) in both the selected K^+- (**c**, **d**) and Na^+-rich (**e**, **f**) buffer solutions.

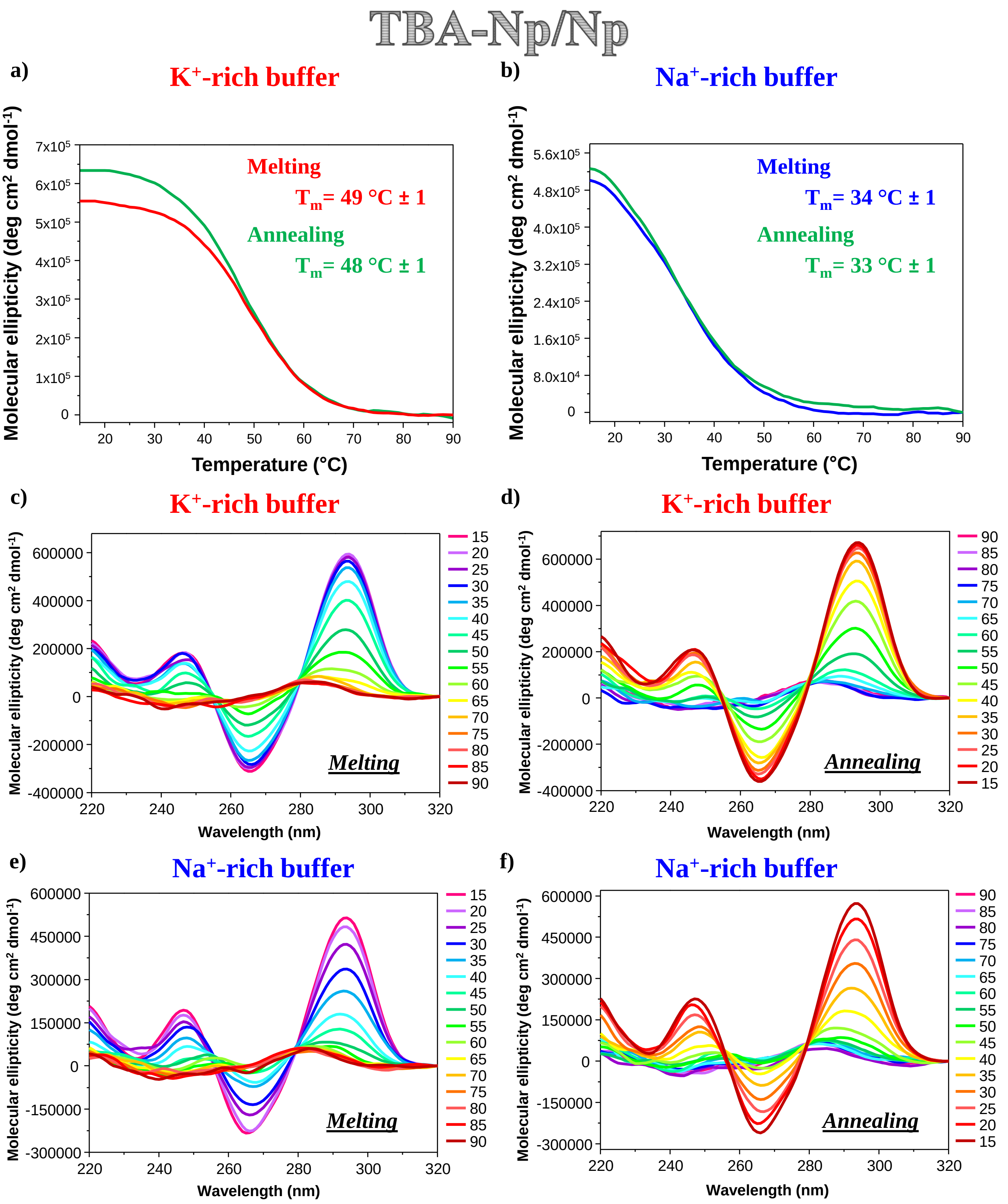

Figure S12. CD analysis of TBA-Np/Np. CD-melting and CD-annealing profiles (**a**, **b**) of **TBA-Np/Np** at 2 μM concentration in both the selected K^+- (**a**) and Na^+-rich (**b**) buffer solutions. The CD curves were obtained following the CD signal at 294 nm in both saline conditions, with a temperature scan rate of 1 °C/min. The overlapped CD spectra of **TBA-Np/Np** were recorded every 5 °C during the melting (**c**, **e**) and cooling processes (**d**, **f**) in both the selected K^+- (**c**, **d**) and Na^+-rich (**e**, **f**) buffer solutions.

TBA-NNp/NNp

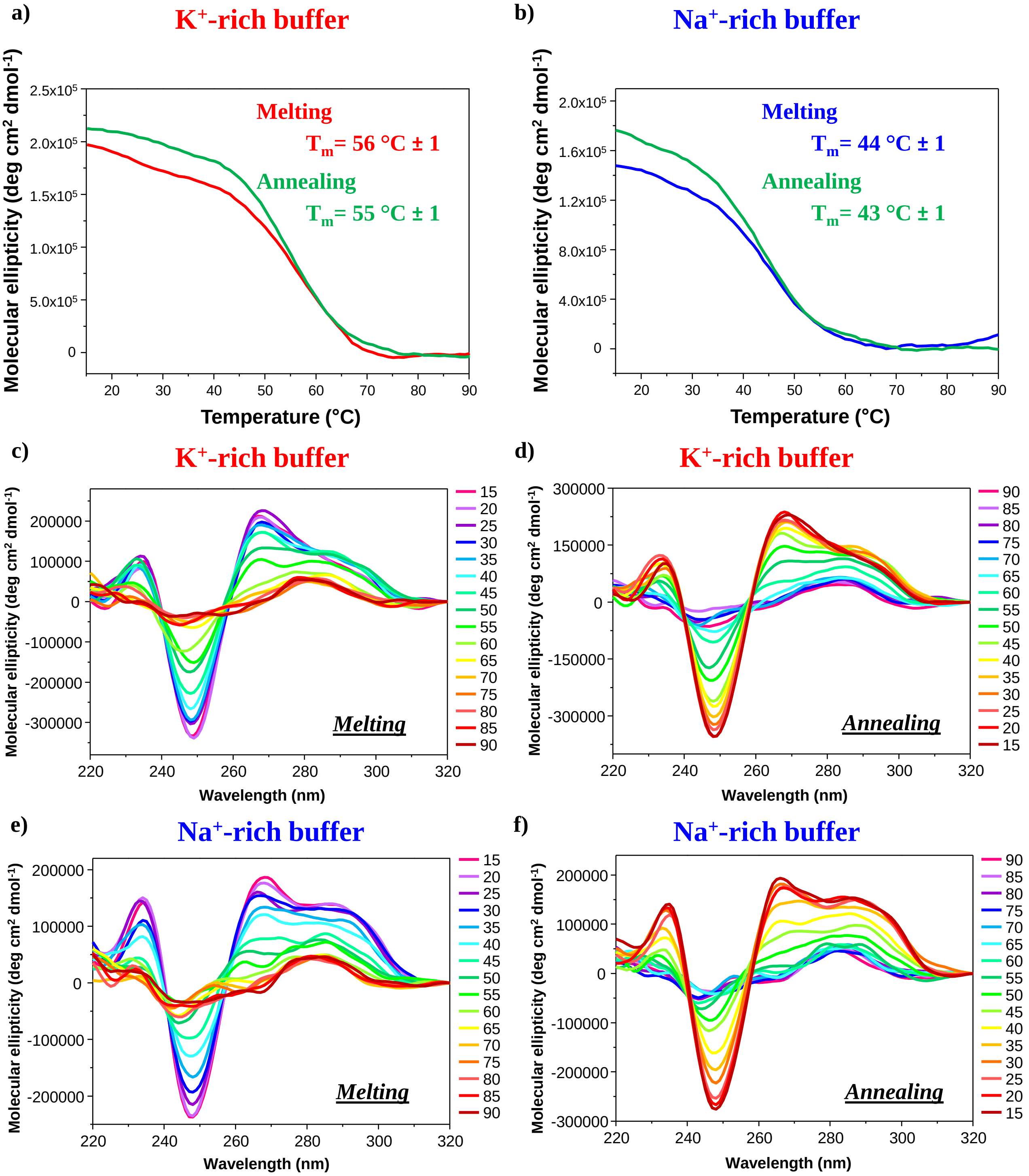

Figure S13. CD analysis of TBA-NNp/NNp. CD-melting and CD-annealing profiles (**a**, **b**) of **TBA-NNp/NNp** at 2 μM concentration in both the selected K^+- (**a**) and Na^+-rich (**b**) buffer solutions. The CD curves were obtained following the CD signal at 268 nm in both saline conditions, with a temperature scan rate of 1 °C/min. The overlapped CD spectra of **TBA-NNp/NNp** were recorded every 5 °C during the melting (**c**, **e**) and cooling processes (**d**, **f**) in both the selected K^+- (**c**, **d**) and Na^+-rich (**e**, **f**) buffer solutions.

Table S2. Standard thermodynamic parameters from van't Hoff analysis.

Aptamer	K^+-rich buffer			Na^+-rich buffer		
	ΔH^0 (kJ mol^{-1})	ΔS^0 (kJ mol^{-1} K^{-1})	ΔG^0_{298K} (kJ mol^{-1})	ΔH^0 (kJ mol^{-1})	ΔS^0 (kJ mol^{-1} K^{-1})	ΔG^0_{298K} (kJ mol^{-1})
**TBA**	-166.3 ± 2.6	-0.51 ± 0.01	-13.6 ± 0.2	-146.6 ± 3.2	-0.47 ± 0.01	-5.7 ± 0.1
**TBA-D**	-140.8 ± 1.1	-0.44 ± 0.01	-9.2 ± 0.1	-128.6 ± 1.7	-0.42 ± 0.01	-3.2 ± 0.1
**TBA-N**	-179.1 ± 4.5	-0.53 ± 0.01	-20.5 ± 0.5	-159.0 ± 1.7	-0.49 ± 0.01	-13.0 ± 0.3
**TBA-Np/Dp**	-158.0 ± 1.7	-0.49 ± 0.01	-13.2 ± 0.2	-135.6 ± 3.2	-0.43 ± 0.01	-6.3 ± 0.2
**TBA-N/Dp**	-167.3 ± 6.7	-0.50 ± 0.02	-19.4 ± 0.4	-139.1 ± 2.7	-0.43 ± 0.01	-10.7 ± 0.1
**TBA-Np/D**	-147.5 ± 4.6	-0.45 ± 0.01	-14.6 ± 0.5	-136.6 ± 0.1	-0.43 ± 0.02	-9.0 ± 0.8
**TBA-N/D**	-146.8 ± 1.0	-0.43 ± 0.01	-19.0 ± 0.1	-134.8 ± 1.9	-0.41 ± 0.01	-11.4 ± 0.2
**TBA-NNp/DDp**	-147.1 ± 4.1	-0.44 ± 0.01	-16.8 ± 0.4	-118.4 ± 2.0	-0.37 ± 0.01	-8.2 ± 0.3
**TBA-NN/DD**	-140.5 ± 1.5	-0.42 ± 0.01	-15.4 ± 0.1	-105.9 ± 1.3	-0.33 ± 0.01	-6.5 ± 0.1
**TBA-Np/Np**	-136.9 ± 1.6	-0.42 ± 0.01	-10.6 ± 0.1	-104.9 ± 0.1	-0.34 ± 0.01	-3.6 ± 0.1

Standard thermodynamic parameters as derived from van't Hoff analysis (ΔH^0, ΔS^0 and ΔG^0 calculated at 298 K) for the unfolding process of unmodified TBA and its modified analogues, followed by CD spectroscopy in the selected buffer solutions.

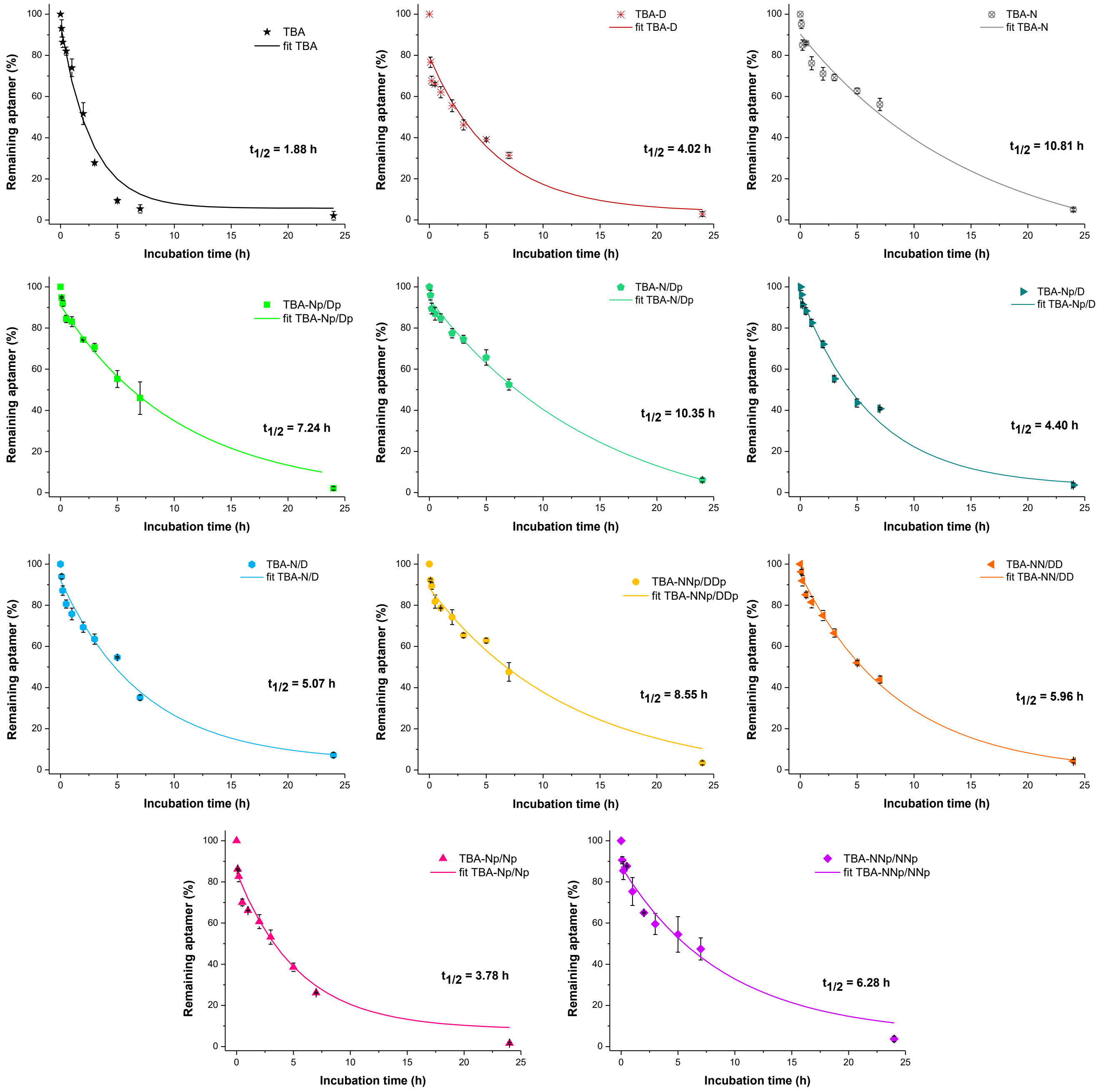

Figure S14. Nuclease resistance experiments on the TBA analogues. Enzymatic resistance experiments performed on TBA and its analogues incubated in 80% fetal bovine serum (FBS) as monitored by denaturing 20% polyacrylamide gel electrophoresis up to 24 h (time points: 0, 0.1, 0.2, 0.5, 1, 2, 3, 5, 7 and 24 h). Intensity of each oligonucleotide band on the gel is expressed as percentage of the remaining aptamer with respect to the initial one (untreated oligonucleotide) for all the analyzed time points. Data are reported as mean values ± SD (error bars) for three independent determinations. Obtained values were also fitted with an equation for first order kinetics (lines) allowing the calculation of the half-life in serum of each aptamer ($t_{1/2}$).

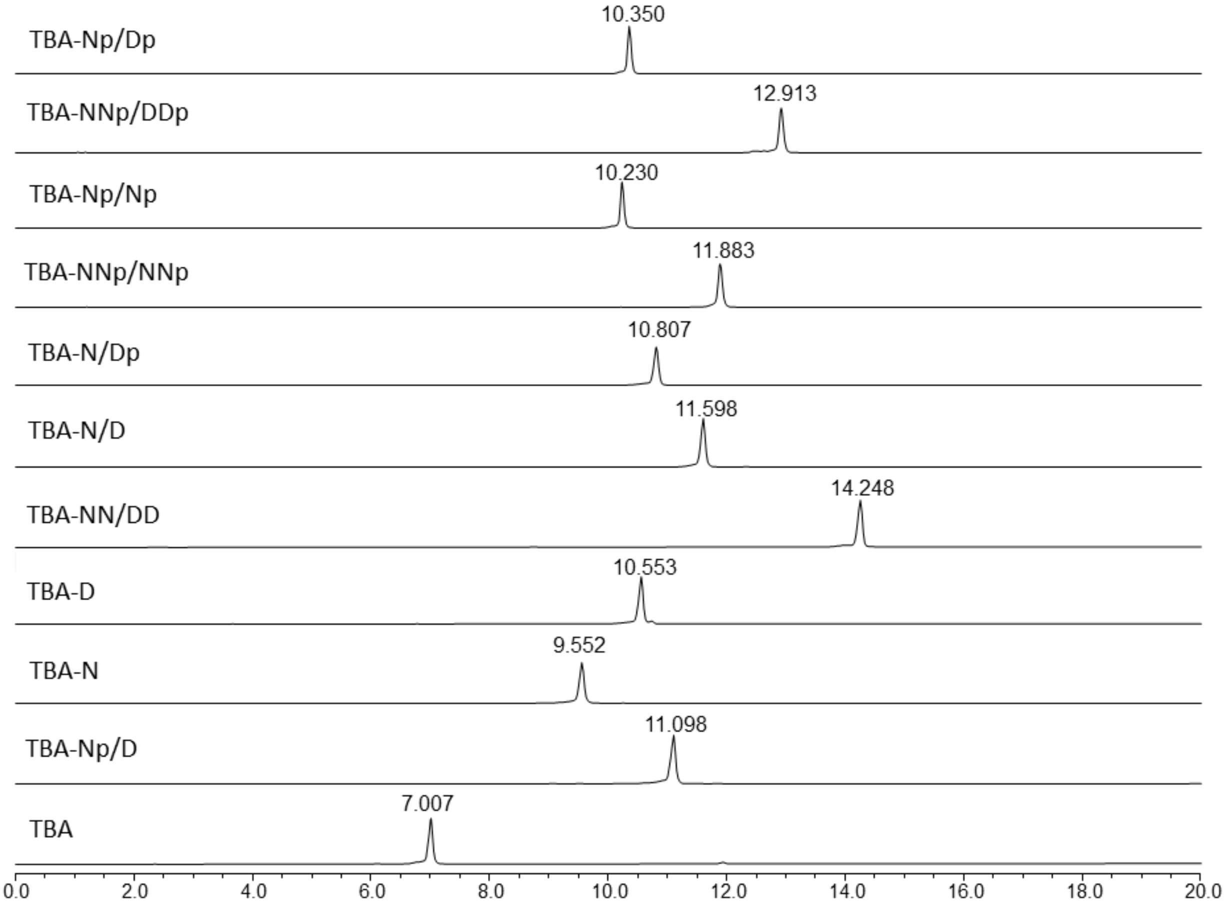

Figure S15. Comparative reverse phase C18 HPLC analysis of the TBA analogues. Column Nucleodur 100-3 C18 ec, length 75 mm, diameter 4.6 mm (Macherey-Nagel). Linear gradient 1 mL/min, from 1% to 40% of CH_3CN in 50 mM TEAAc buffer pH 7 60 °C, over 20 min.

Table S3. Summary of aptamers properties.

Aptamers	Tm (°C) ± 1				Serum resistance		anticoagulation activity		Relative lipophilicty	
	Na^+ rich	◎Tm[a]	K^+ rich	◎Tm[a]	t1/2 (h)	N[b]	score	N[c]	Rt (min)	N[d]
TBA	36	-	51	-	1.9	1.0	8.0	1.0	7.0	1.0
TBA-D	31	-5	45	-6	4.0	2.1	Nd	-	10.6	1.5
TBA-N	51	15	64	13	10.8	5.7	Nd	-	9.6	1.4
TBA-Np/Dp	38	2	51	0	7.2	3.8	7.3	0.9	10.4	1.5
TBA-N/Dp	48	12	62	11	10.4	5.5	7.1	0.9	10.8	1.5
TBA-Np/D	42	6	57	6	4.4	2.3	6.3	0.8	11.1	1.6
TBA-N/D	51	15	68	17	5.1	2.7	2.7	0.3	11.6	1.7
TBA-NNp/DDp	44	8	62	11	8.5	4.5	9.3	1.2	12.9	1.8
TBA-NN/DD	42	6	61	10	6.0	3.2	2.8	0.4	14.3	2.0
TBA-Np/Np	34	-2	49	-2	3.8	2.0	9.0	1.1	10.2	1.5
TBA-NNp/NNp	44	8	56	5	6.3	3.3	2.3	0.3	11.9	1.7

[a]ΔTm relative to TBA calculated as Tm of aptamer - Tm of TBA, [b]Normalized data calculated as $t_{1/2}$ of aptamer / $t_{1/2}$ of TBA, [c]Normalized data calculated as score of aptamer / score of TBA, [d]Normalized data calculated as Retention time (Rt) of aptamer / Rt of TBA on a C_{18} reverse phase column. Nd: not determined

Table S4. Reverse phase C18 HPLC and MALDI-TOF MS analysis of the TBA analogues.

Compounds	Retention time (min)[a]	MALDI Calcd	-TOF MS[b] Found	Scale (μmol)	Quantity obtained (nmol)	Yield (%)
TBA-Np/Dp	10.35	5783.83	5783.77	1.5	148.5	9.9
TBA-N/D	11.60	5507.71	5507.22	1.5	92.4	6.2
TBA-Np/D	11.10	5645.77	5646.88	1.5	88.8	5.9
TBA-N/Dp	10.81	5645.77	5646.72	1.2	335.5	28.0
TBA-NNp/DDp	12.91	6566.46	6565.59	1.5	246.3	16.4
TBA-NN/DD	14.25	6290.34	6290.28	1.5	68.7	4.6
TBA-D	10.55	5063.37	5064.18	1.2	199.8	16.7
TBA-Np/Np	10.23	5889.87	5888.68	1.2	101.5	8.5
TBA-NNp/NNp	11.88	6778.54	6779.39	1.2	94.5	7.9
TBA-N	9.55	5169.41	5170.29	1.2	278.5	23.2

[a]Linear gradient 1 mL/min, from 1% to 40% of CH_3CN in 50 mM TEAAc buffer pH 7 60 °C, over 20 min, [b]$[M-H]^-$

MALDI-TOF and RP C_{18} HPLC of TBA-Np/Dp

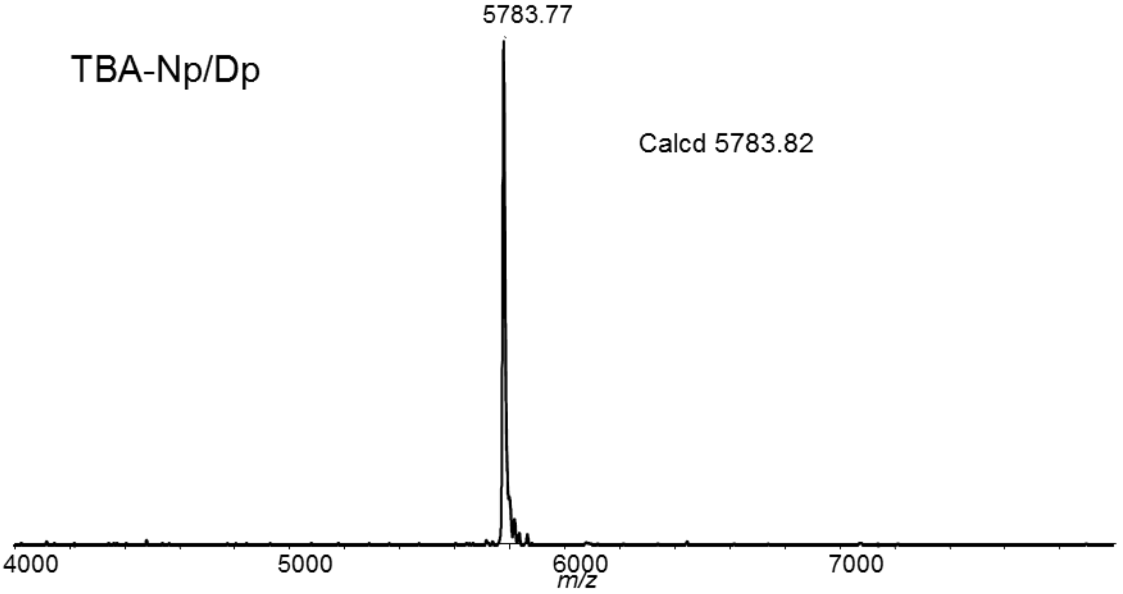

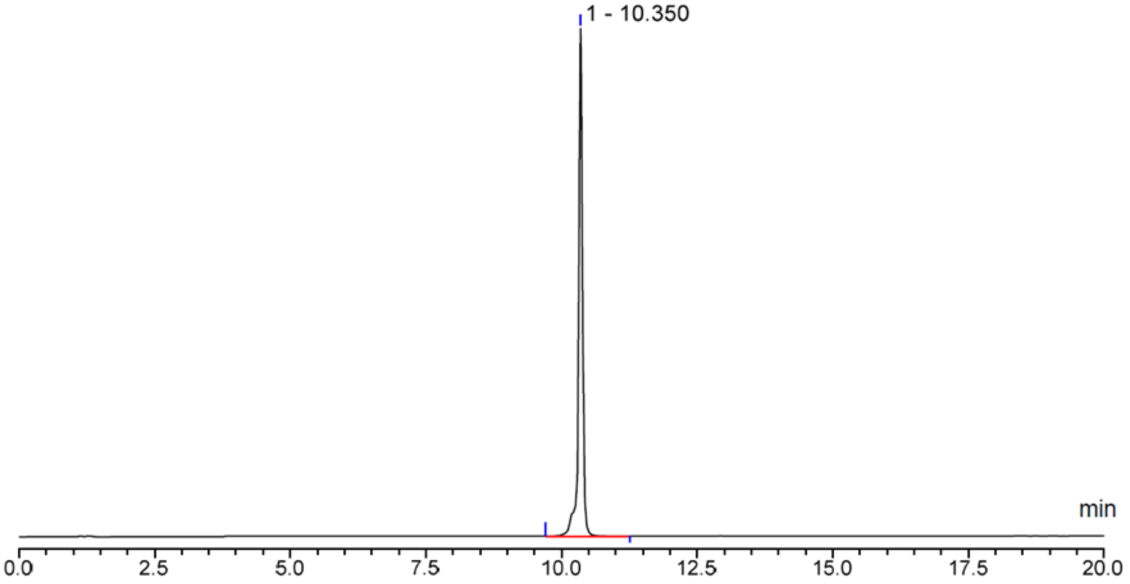

MALDI-TOF and RP C_{18} HPLC of TBA N/D

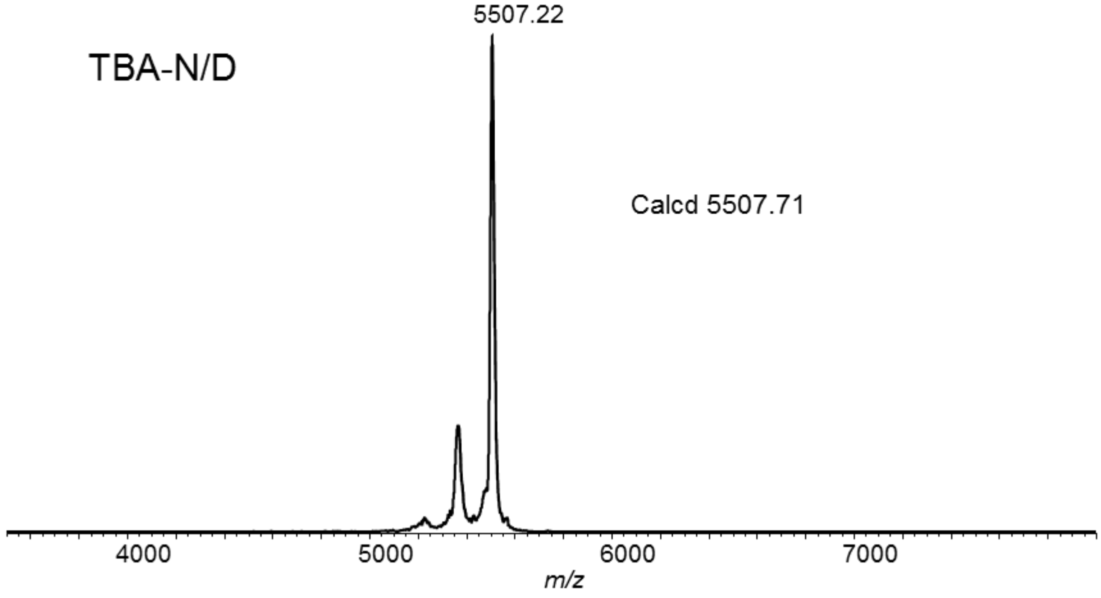

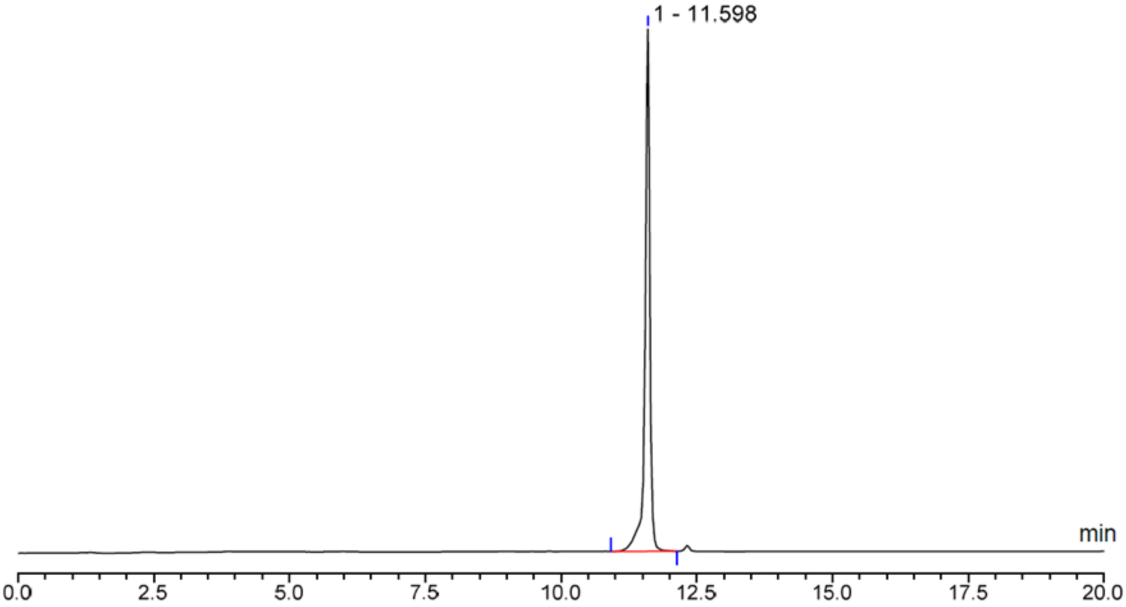

MALDI-TOF and RP C_{18} HPLC of TBA-Np/D

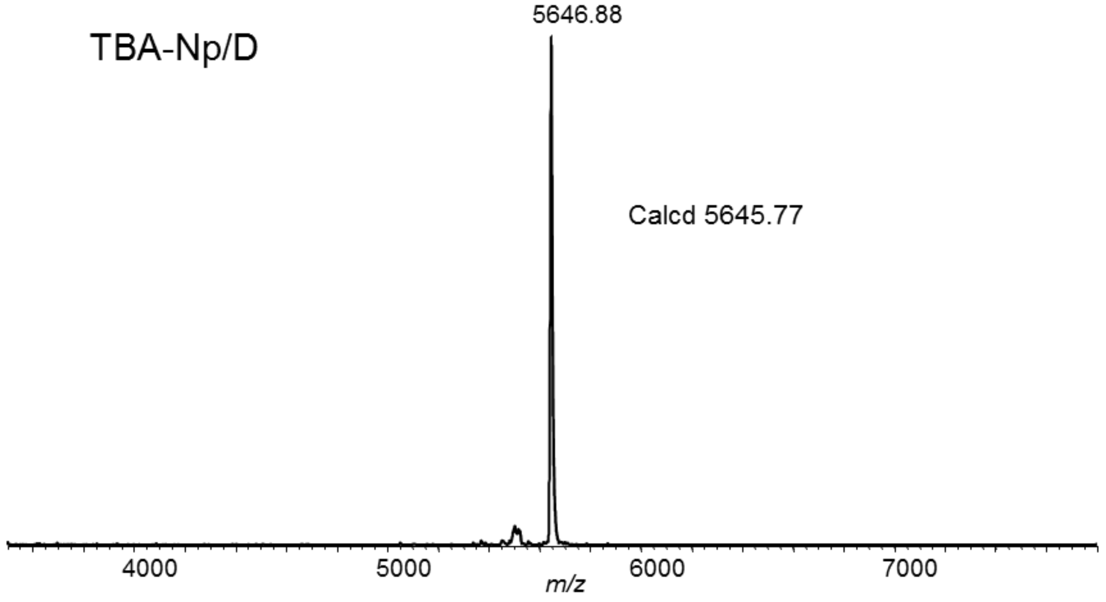

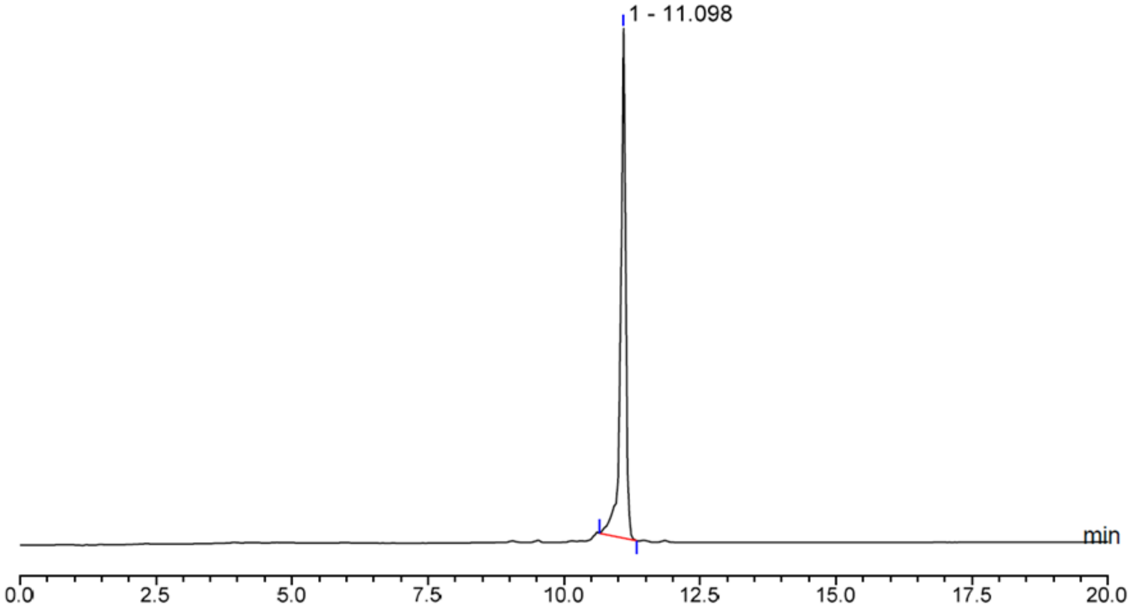

MALDI-TOF and RP C_{18} HPLC of TBA-N/Dp

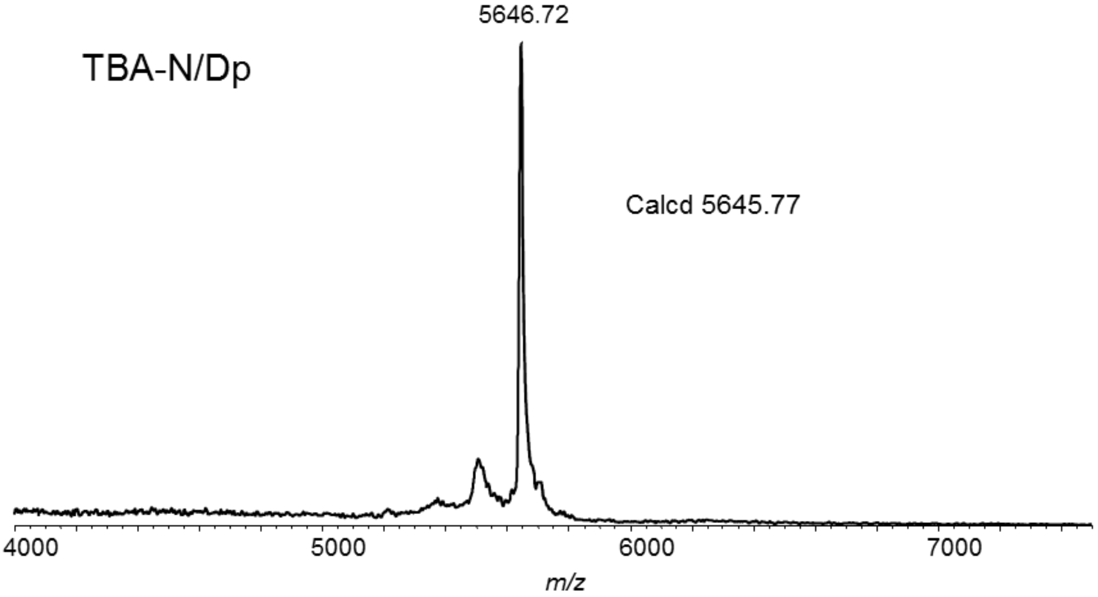

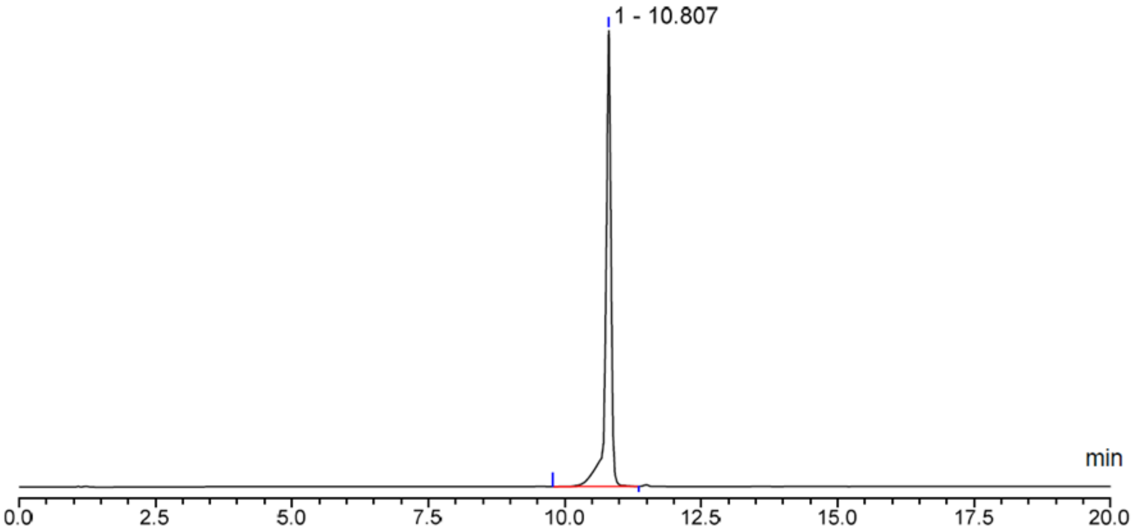

MALDI-TOF and RP C_{18} HPLC of TBA-NNp/DDp

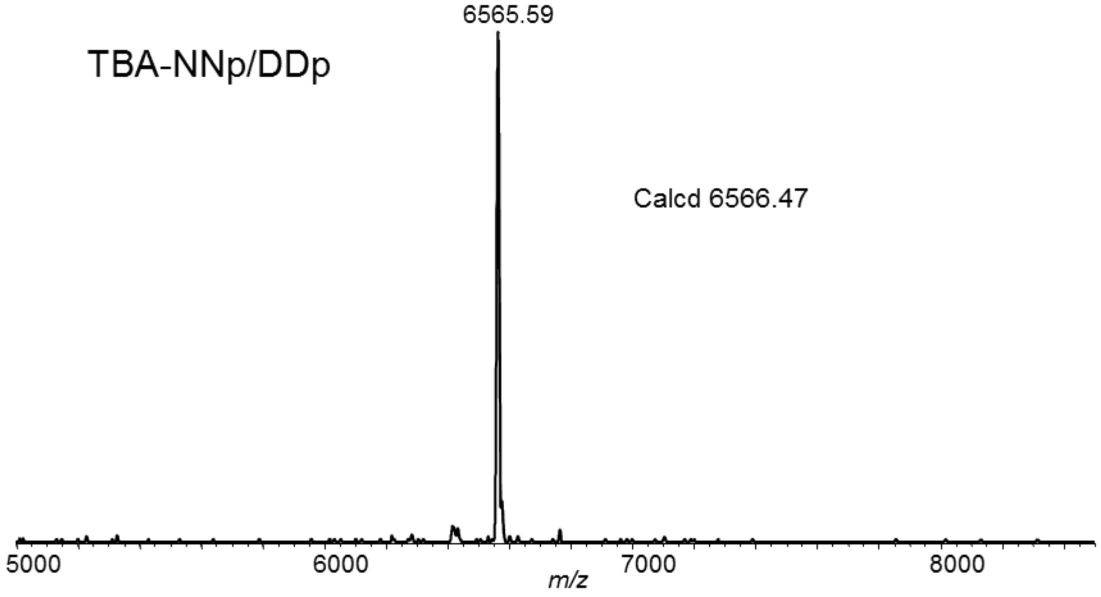

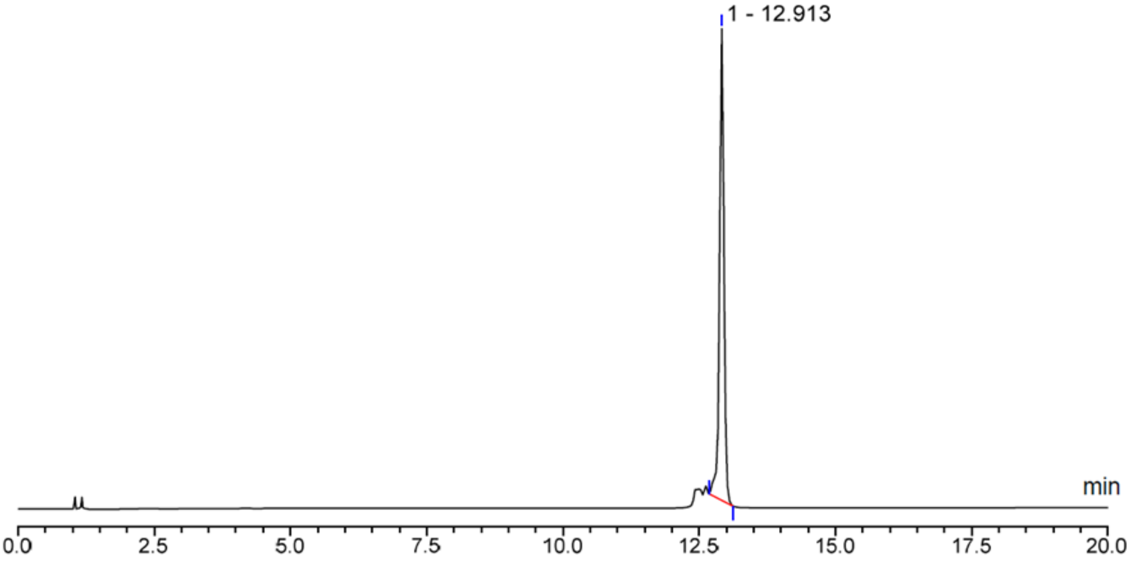

MALDI-TOF and RP C_{18} HPLC of TBA-NN/DD

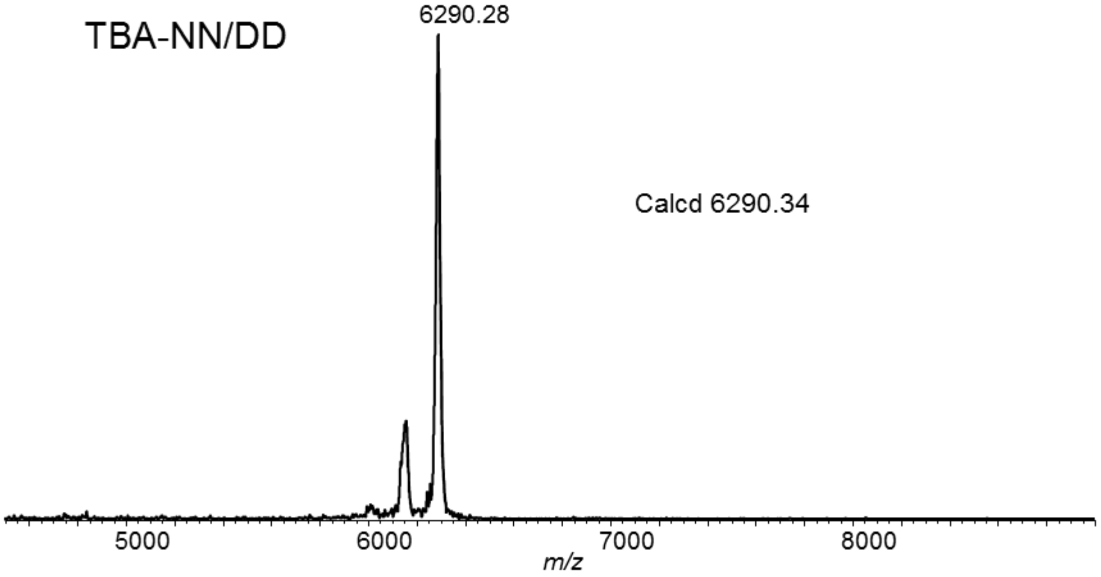

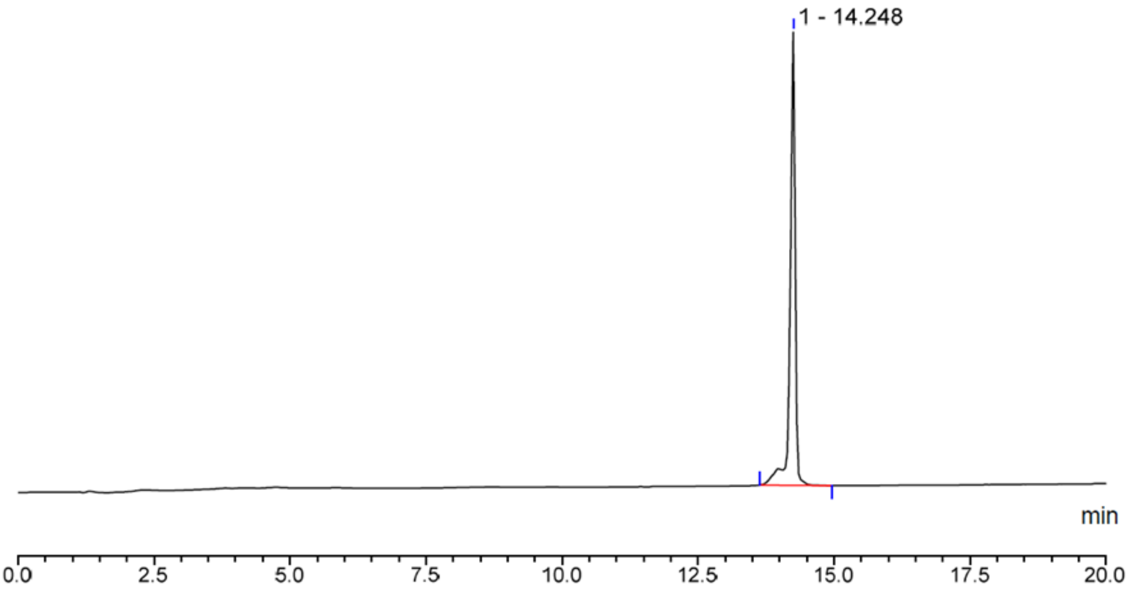

MALDI-TOF and RP C_{18} HPLC of TBA-D

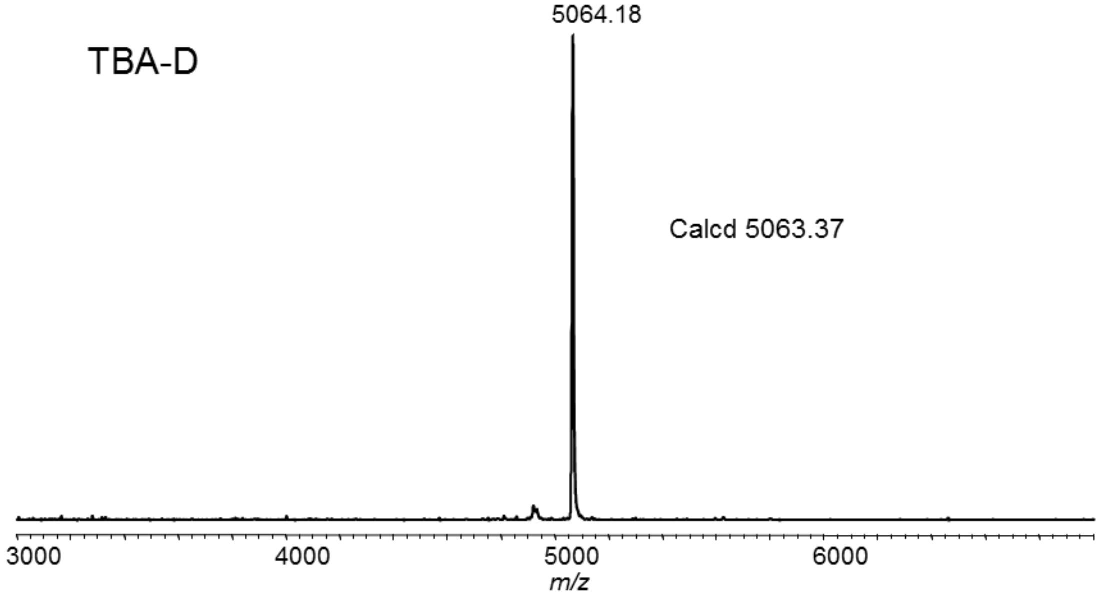

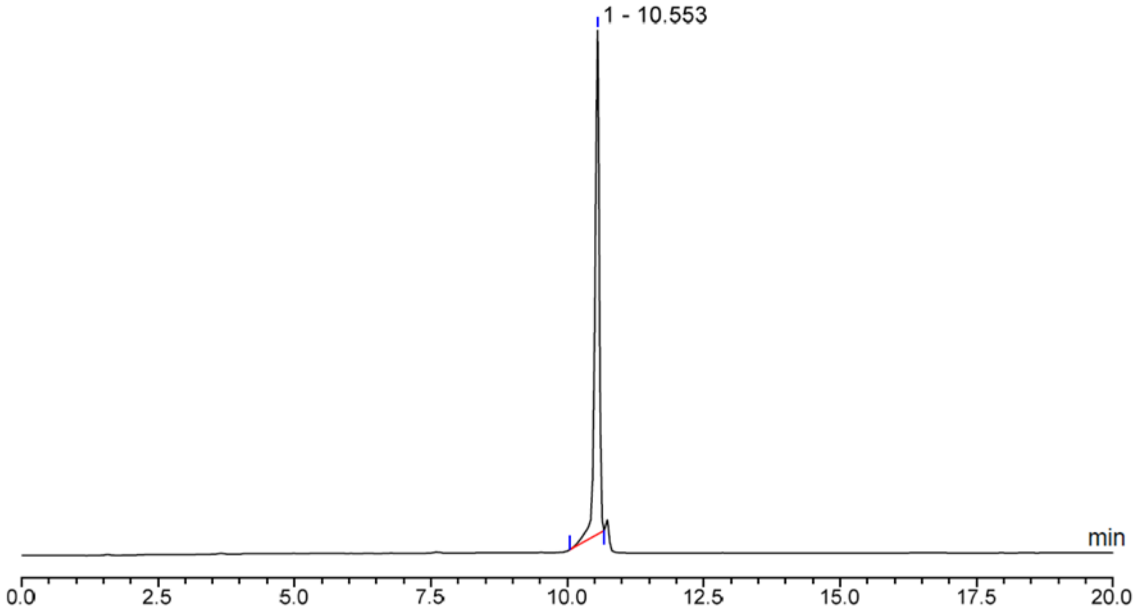

MALDI-TOF and RP C_{18} HPLC of TBA-Np/Np

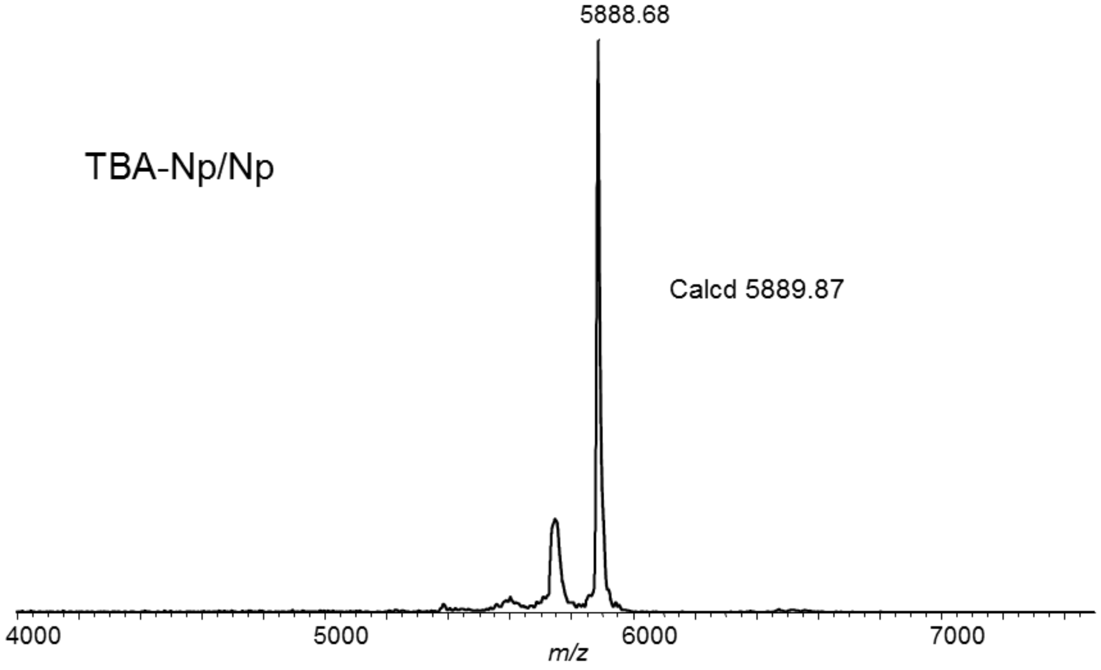

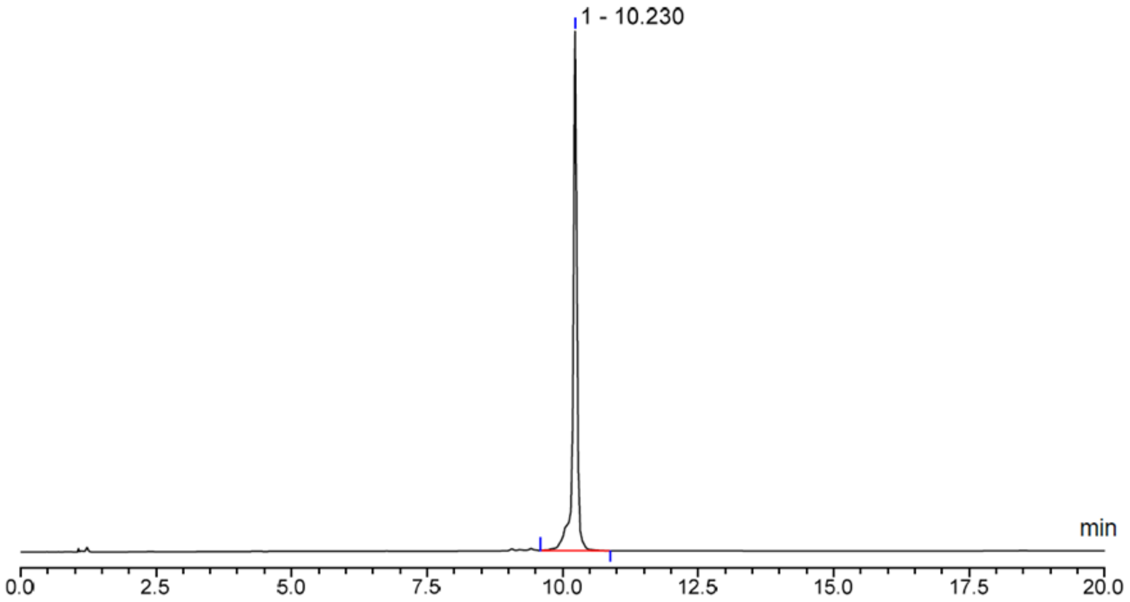

MALDI-TOF and RP C_{18} HPLC of TBA-NNp/NNp

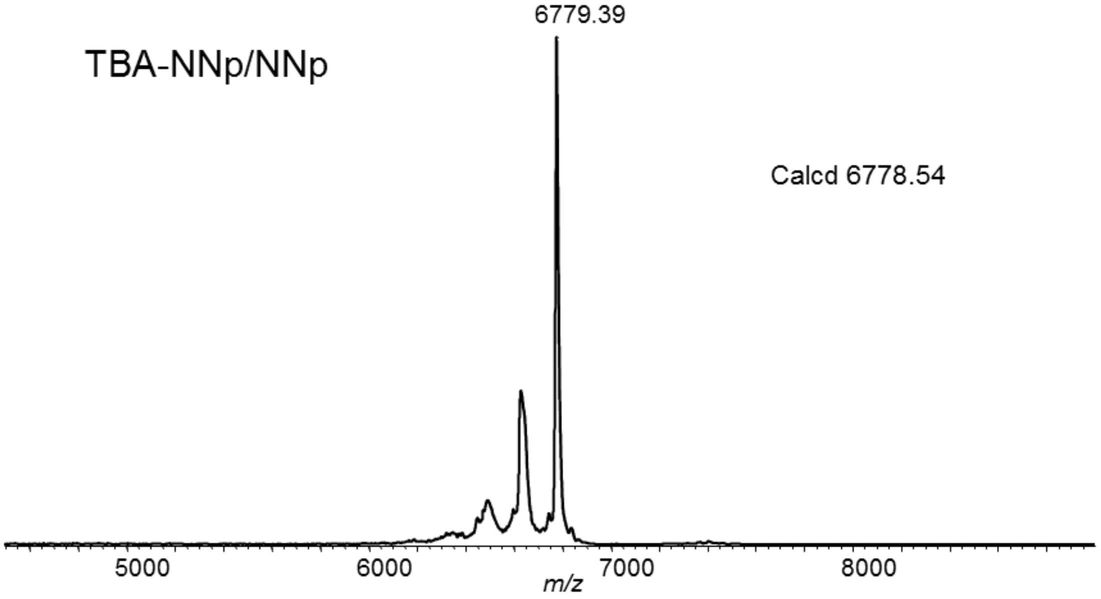

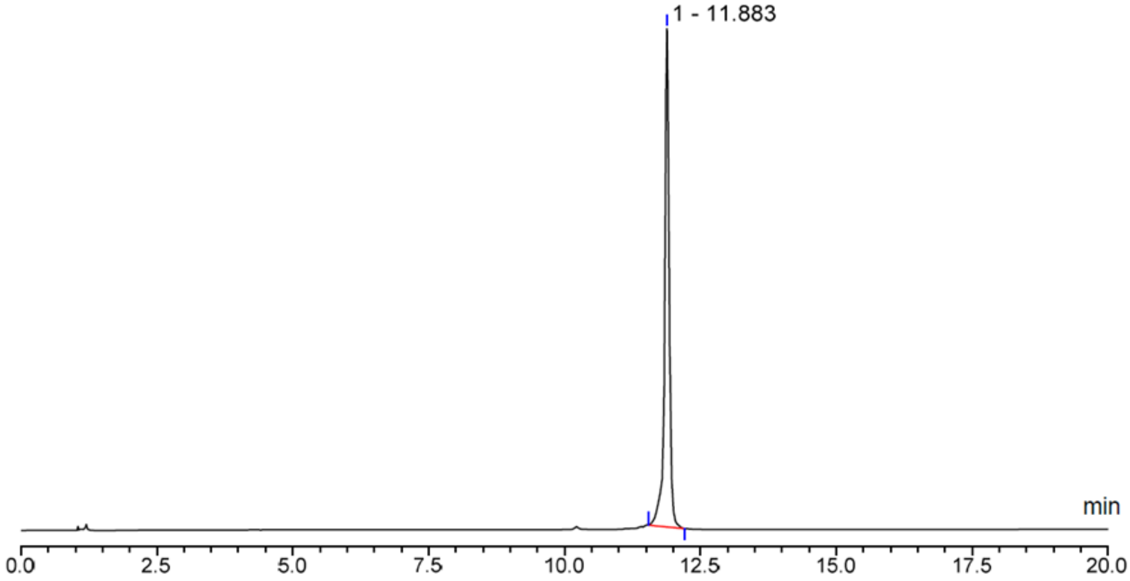

MALDI-TOF and RP C_{18} HPLC of TBA-N
Calcd 5169.41

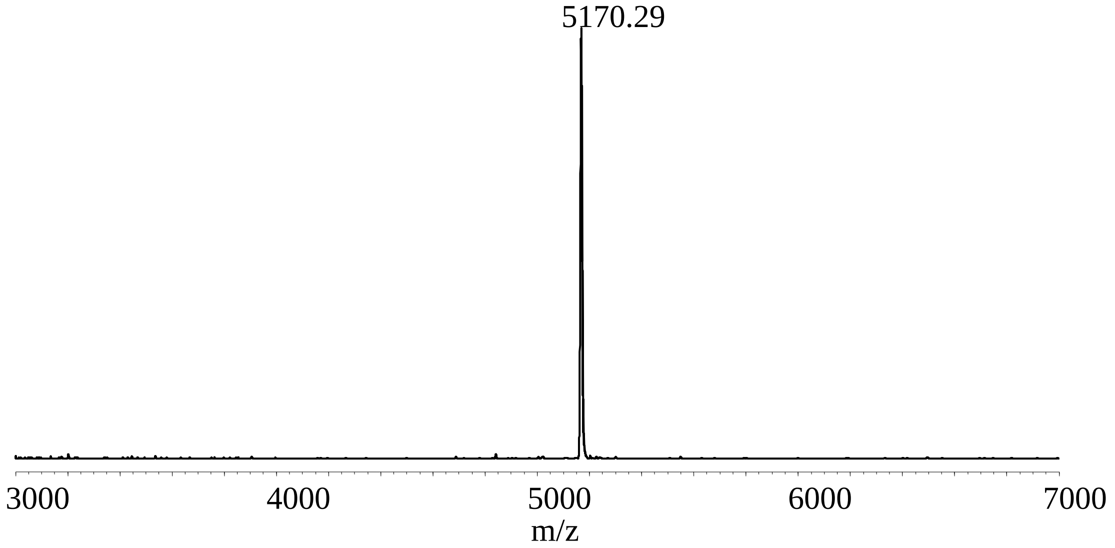

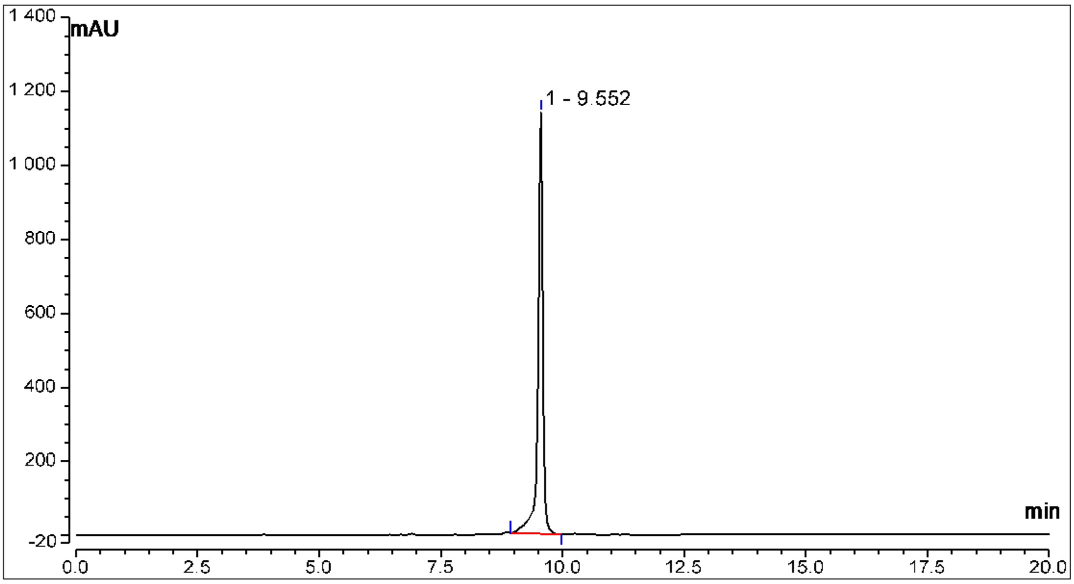

References:

1. Karsisiotis,A.I., Hessari,N.M.A., Novellino,E., Spada,G.P., Randazzo,A. and Webba da Silva,M. (2011) Topological characterization of nucleic acid G-quadruplexes by UV absorption and circular dichroism. *Angew. Chem. - Int. Ed. Eng.*, **50**, 10645–10648.
2. Del Villar-Guerra,R., Trent,J.O. and Chaires,J.B. (2018) G-quadruplex secondary structure obtained from circular dichroism spectroscopy. *Angew. Chem. - Int. Ed. Eng.*, **57**, 7171–7175.
